# Molecular classification of prostate adenocarcinoma by the integrated somatic mutation profiles and molecular network

**DOI:** 10.1038/s41598-017-00872-8

**Published:** 2017-04-07

**Authors:** Lei Yang, Shiyuan Wang, Meng Zhou, Xiaowen Chen, Wei Jiang, Yongchun Zuo, Yingli Lv

**Affiliations:** 1 College of Bioinformatics Science and Technology, Harbin Medical University, Harbin, 150081 China; 2 The Key Laboratory of Mammalian Reproductive Biology and Biotechnology of the Ministry of Education, Inner Mongolia University, Hohhot, 010021 China

## Abstract

Prostate cancer is one of the most common cancers in men and a leading cause of cancer death worldwide, displaying a broad range of heterogeneity in terms of clinical and molecular behavior. Increasing evidence suggests that classifying prostate cancers into distinct molecular subtypes is critical to exploring the potential molecular variation underlying this heterogeneity and to better treat this cancer. In this study, the somatic mutation profiles of prostate cancer were downloaded from the TCGA database and used as the source nodes of the random walk with restart algorithm (RWRA) for generating smoothed mutation profiles in the STRING network. The smoothed mutation profiles were selected as the input matrix of the Graph-regularized Nonnegative Matrix Factorization (GNMF) for classifying patients into distinct molecular subtypes. The results were associated with most of the clinical and pathological outcomes. In addition, some bioinformatics analyses were performed for the robust subtyping, and good results were obtained. These results indicated that prostate cancers can be usefully classified according to their mutation profiles, and we hope that these subtypes will help improve the treatment stratification of this cancer in the future.

## Introduction

Prostate cancer is the most non-cutaneous common cancer in males and one of the leading causes of cancer-related deaths worldwide. The incidence and mortality of prostate cancer exhibit a remarkable variety in different parts of the world, and they are highest in the western world^1^. It is estimated that 220,800 men were diagnosed with prostate cancer and that 27,540 will die of the disease in 2015 in the United States^2^. Several demographic, clinical and genetic factors, including age, race, family history, genetic susceptibility, and prostate-specific antigen (PSA) level, have contributed to the high incidence of prostate tumors^3^. Despite the high incidence of these carcinomas, prostate cancer is often an indolent cancer. Many patients who have indolent prostate cancer will remain asymptomatic for many years after diagnosis, and many others can even live for more than ten years with organ-confined disease^4^. With the emergence and application of new genomic technologies, such as next-generation sequencing and microarray analyses, more molecular and genetic profiles of prostate adenocarcinomas have been generated in recent years. Based on these profiles, we found that prostate adenocarcinomas exhibit a remarkable biological heterogeneity, including alterations of somatic copy number, point mutations, and structural rearrangements, and these genetic heterogeneities may underlie the high variability of clinical outcomes in prostate adenocarcinomas^5–10^. Given the tremendous biological heterogeneity of prostate tumors, it is critical to determine the appropriate treatment for patients diagnosed with prostate adenocarcinoma. Therefore, understanding the biological heterogeneity of prostate adenocarcinomas is one of the fundamental goals of cancer informatics, and some studies have shown that classification of prostate cancers into clinically and biologically meaningful subtypes can provide more precise outcome predictions, additional information on the selection of optimal therapies, and a better understanding of the heterogeneity^1, 11–15^.

Because the classification of cancers into clinically meaningful subtypes provides insights into the biological properties responsible for tumor progression and guides treatment and prognosis more precisely, more molecular profiles are being used to subtype all types of cancers. Currently, large-scale genomics projects, including The Cancer Genome Atlas (TCGA) Research Network, are producing molecular profiles for thousands of malignancies, rendering the molecular subtyping of distinct malignancies possible. In the past few years, gene expression data have been used to stratify different molecular subtypes of malignancies by several recent studies^11, 12, 16, 17^. Based on gene expression profiles of 26000 genes, Lapointe *et al*. first distinguished prostate cancers from normal samples, and further identified three subtypes of prostate cancers by using unsupervised hierarchical clustering. They also found two genes can be used as surrogate markers for tumor subtypes for predicting tumor recurrence^16^. Markert *et al*., analyzed a microarray dataset of 281 prostate cancers, and five distinct molecular subtypes were identified by unsupervised clustering. They found that the first subtype was characterized by poor survival outcome, the second subtype was characterized by intermediate survival outcome, and three subtypes were characterized by benign outcome. They also validated their stratification on an independent dataset of 150 tumor samples^17^. In the work of Tomlins *et al*., they analyzed the gene expression profiles of prostate cancer for 1577 patients. Three distinct molecular subtyping, including m-ERG^+^ subtype, m-ETS^+^ subtype, and m-SPINK1^+^ subtype were identified in their study, and these molecular subtypes of prostate cancer were supported by transcriptomic and clinical analysis^12^. In addition, genomics data from multiple assay platforms, including mRNAseq, miRNA-seq, and DNA methylation data, have been integrated by TCGA to stratify more than ten distinct malignancies, and the stratification results have shown that each cancer type can be divided into three or four molecular subtypes^1, 18–27^. However, the somatic mutation profiles were seldom used by those studies in the area of tumor subtyping because those data are extremely sparse and rarely shared across patients; thus, they could not be easily used like other molecular profiles^28–32^. Somatic mutations often disrupt the function of mutated genes, providing insights into the mechanisms of tumorigenesis and tumor progression; therefore, stratification tumors with somatic mutation profiles may provide more effective clinical guidance^33^. Indeed, some prior attempts integrated somatic mutation profiles and molecular networks to stratify various distinct malignancies into meaningful molecular subtypes^28, 29^. However, until now, no attempt has been made to use somatic mutation profiles to stratify prostate cancers.

In this study, by integrating somatic mutation profiles and a protein-protein interaction network, we constructed smoothed mutation profiles in 498 prostate adenocarcinoma samples from TCGA. We created a Graph-regularized Nonnegative Matrix Factorization (GNMF)^34^ input matrix for 498 tumor samples using the top 500 most variant genes that were selected by ranking smoothed mutation profiles with the coefficient of variation across the samples. The GNMF was applied to stratify distinct molecular subtypes of prostate adenocarcinoma with subtype sizes ranging from k = 2 to k = 9. The statistical tests demonstrated that these different sizes of subtypes were associated with most of the clinical and pathological characteristics. Specifically, the subtypes with k = 3, which had the highest cophenetic coefficients, were defined as the robust subtypes^35^. The Elastic Net algorithm^36^ was used to classify the robust subtypes by using the top 500 most variant genes as the input parameters, and good predictive results were obtained in this study. In addition, the biomarker genes of each molecular subtype were selected by the Elastic Net algorithm. The functional annotation tool DAVID^37^ was also used for the enrichment analysis of these biomarker genes, and some cancer-related KEGG pathways and GO terms were detected by this bioinformatics tool. As demonstrated by a series of recent publications^38–49^ in compliance with Chou’s 5-step rule^50^, to establish a really useful sequence-based statistical predictor for a biological or biomedical system, we should follow the following five guidelines: (a) construct or select a valid benchmark dataset to train and test the predictor; (b) formulate the biological sequence samples with an effective mathematical expression that can truly reflect their intrinsic correlation with the target to be predicted; (c) introduce or develop a powerful algorithm (or engine) to operate the prediction; (d) properly perform cross-validation tests to objectively evaluate the anticipated accuracy of the predictor; (e) establish a user-friendly web-server for the predictor that is accessible to the public. Below, we are to describe how to deal with these steps one-by-one.

## Results

### Analysis of mutation patterns in prostate adenocarcinoma

**Figure 1:**
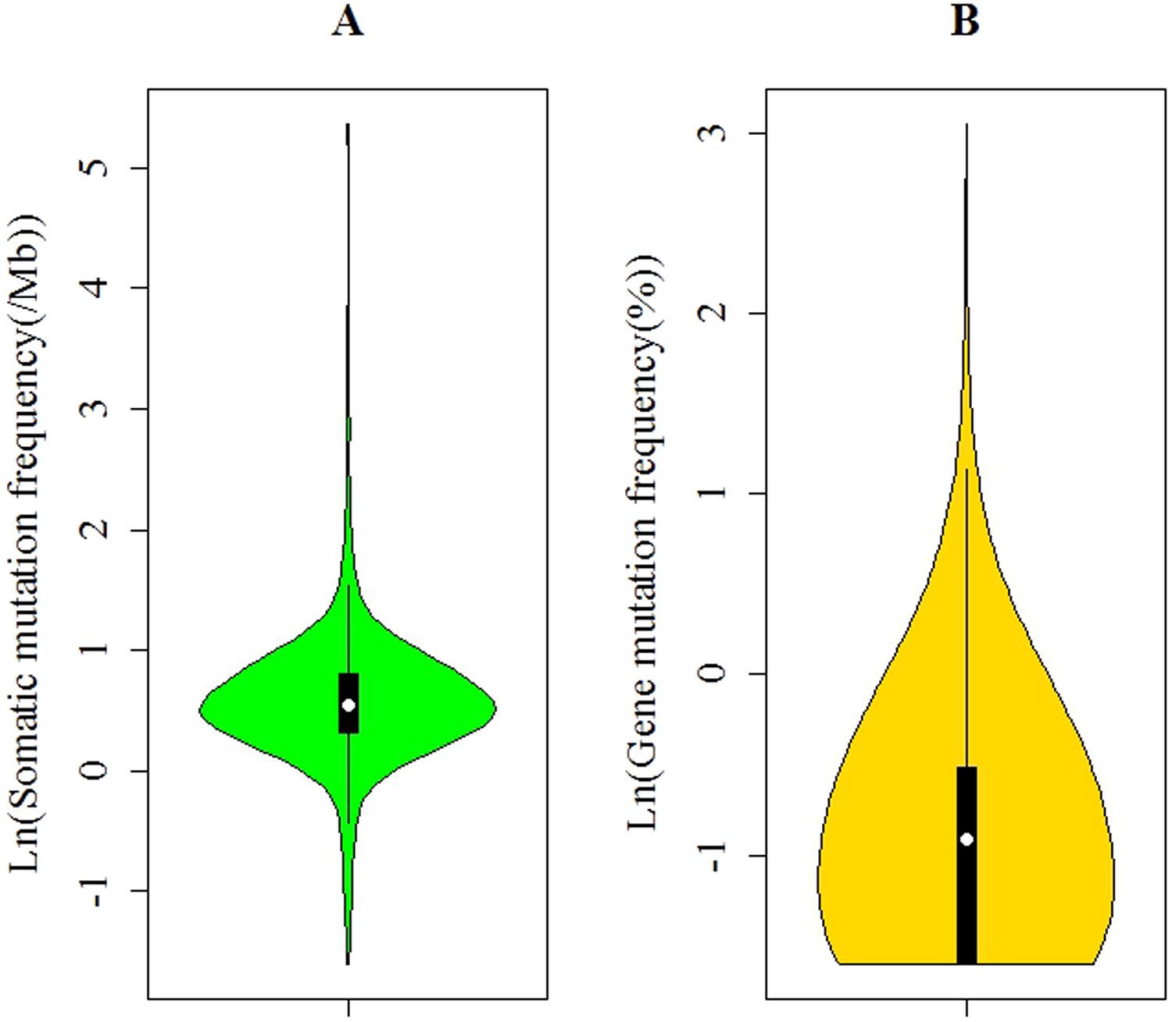
The violin plots for (**A**) the frequency of genes mutated per patient in the entire cohort and (**B**) the frequency of mutations per gene in the entire cohort. Figure 1A illustrated that the somatic mutation frequencies varied more than three orders of magnitude across different patients. Figure 1B illustrated that only a small number of genes mutated at high frequencies, most genes mutated at intermediate or low frequencies.

**Figure 2:**
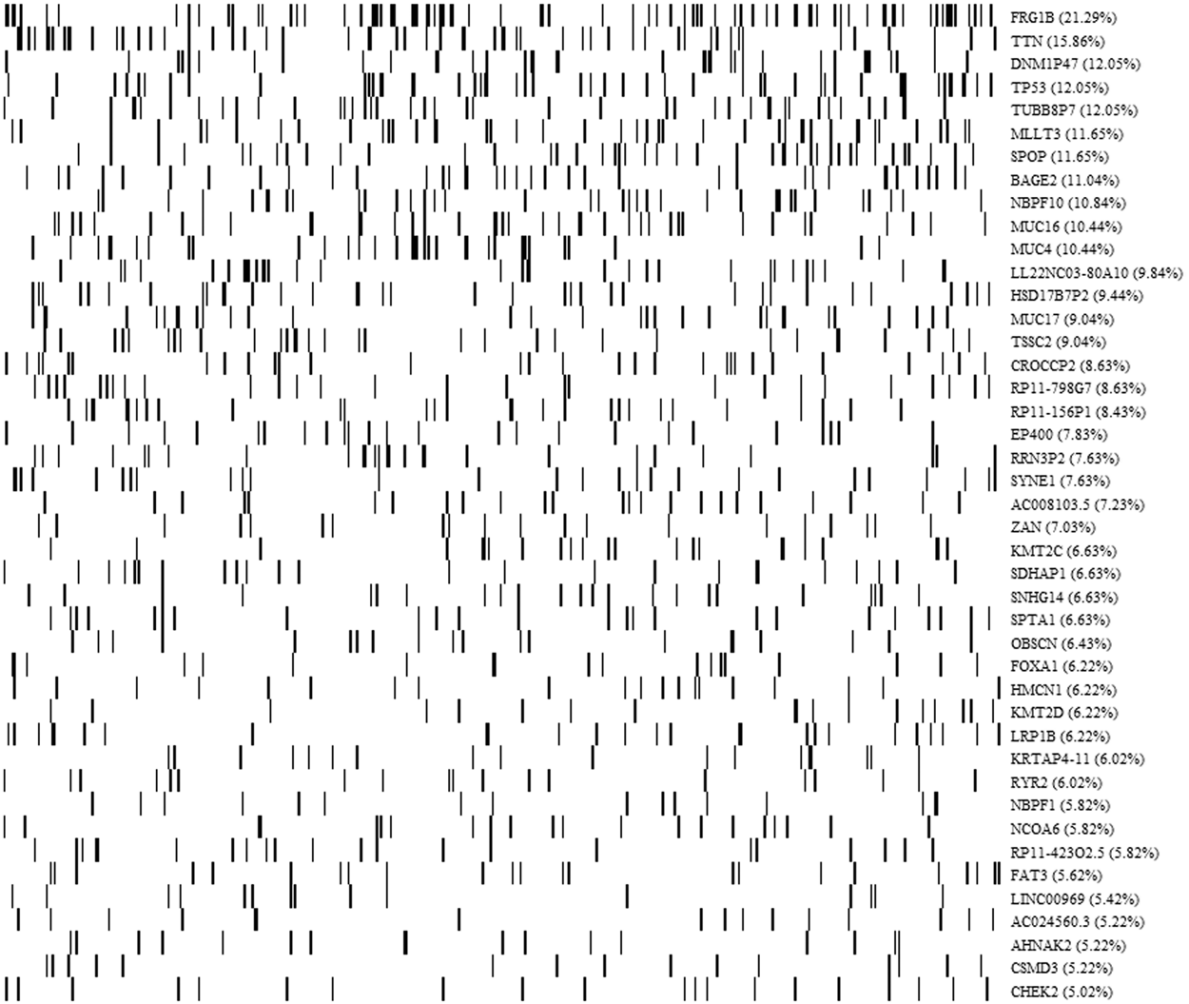
The somatic mutation profiles of 498 tumor patients. In this figure, only the genes that was mutated in >5% of samples were shown. In this figure, we found that some well-known cancer genes, including FRG1B, TTN, TP53, SPOP and FOXA1 exhibit the higher mutation frequency than other mutated genes in all samples.

TCGA has used the latest sequencing and analysis methods to identify somatic mutations in more than twenty types of tumor. In this study, to explore the feasibility of a comprehensive understanding of mutated genes, we analyzed somatic point mutations in exome sequences from TCGA for 498 prostate adenocarcinoma samples. To compute the somatic mutation frequency, we assumed that a simplified exome comprises 20000 genes, each with the same coding length of 1500 nucleotides, as performed in the work of TCGA^30–32^. Surprisingly, the analysis of the prostate adenocarcinoma revealed that the somatic mutation frequencies vary more than three orders of magnitude (from 0.2 per megabase (Mb) to 214.8 per Mb) across patients within a cancer type (Fig. 1A), consistent with the study of the mutational heterogeneity of approximately 3000 samples^32^. This might be due to heterogeneity in the mutational processes in cancer. Second, after analyzing the total mutation frequency of each sample, we also analyzed the proportion of each mutated gene detected in the total samples. We can also clarify that although some genes mutated in >10% of samples, most genes occurred at intermediate frequencies (1–10%) or even lower (Fig. 1B). In addition, we also plotted the mutational spectrum for the genes that were mutated in >5% of samples and found that the mutational spectrum also varied sharply across mutated genes (Fig. 2). It was very common for clinically identical patients to share no more than a single mutation. Notably, FRG1B (FSHD region gene 1 family, member B) was mutated in 21.29% of samples, exhibiting the highest mutation frequency in this study. In particular, some well-known cancer genes, including TTN (mutated in 15.86% of samples) and TP53 (mutated in 12.05% of samples), were identified. In addition, we identified some genes previously known to be associated with prostate adenocarcinoma, including SPOP, a substrate-binding subunit of a cullin-based E3 ubiquitin ligase complex, to be mutated in 11.65% of samples, and FOXA1, known as hepatocyte nuclear factor 3-alpha (HNF-3A), to be mutated in 6.22% of samples.

### Classification of prostate adenocarcinoma samples

Based on our analysis of mutation patterns in prostate adenocarcinoma, we can conclude that the somatic mutations of prostate adenocarcinoma were remarkably heterogeneous, and the somatic mutation profiles were extremely sparse, with typically fewer than 100 mutated bases in an entire exome. Unlike other data types, such as expression and methylation, the somatic mutations were not assigned a quantitative value for every patient. Thus, classifying the prostate adenocarcinoma samples based only on the mutation profile was very challenging. In this study, by integrating the somatic mutation profiles with the molecular network, these problems can be largely overcome. For each patient, a gene was coded as 1 if this gene had at least one somatic mutation or 0 if no mutation occurred in this gene. Thus, the mutation profile of each patient was represented by a vector of genes marked with 1 or 0. Then, the patient mutation profiles were mapped onto the STRING network. After mapping a patient mutation profile onto the STRING network, the RWRA was applied to spread the influence of each mutation across the STRING network with default parameters. The mutated genes of each patient were used as the source nodes (candidate genes) of the RWRA, and all nodes of the STRING network were then scored. The smoothed mutation profiles of 498 tumor samples were then filtered to eliminate unreliably scored genes and to limit the clustering to relevant genes. To accomplish this, we smoothed the mutation profile variances across the samples that were subsequently used to rank the genes in a descending order. The final filter excluded genes with smaller variability, and the top 500 genes with the highest values of variances across 498 tumor samples were selected. The final smoothed mutation profiles were then median-centered across samples and input into the GNMF algorithm in Matlab 2008a for unsupervised consensus clustering to identify a predefined number of likely subtypes (k = 2, 4, $\cdots$ 9) using the default parameter with lambda = 150. A complete listing of subtype sample membership is available in Supplementary Table S1.

### Clinical relevance of prostate adenocarcinoma subtypes

To determine the clinical relevance of the identified molecular subtypes, the prostate adenocarcinoma subtype associations with the Gleason score, pathologic N, pathologic T, clinical T, and lymph node involvement are investigated in this section. The clinical and pathological characteristics of the 498 patients included in this study are shown in Table 1.

**Table 1:** Clinicopathological characteristics of 498 patient samples.

Parameter		Number	Percentage (%)
Patient age (yrs)*		61	41–78
Follow-up time (days)*		512	1–4602
Gleason score			
	2–6	53	10.64
	7	246	49.40
	8–10	199	39.96
Pathologic N stage			
	N0	346	69.48
	N1	79	15.86
	NA	73	14.66
Pathologic T stage			
	T2	188	37.75
	T3	293	58.84
	T4	11	2.21
	NA	6	1.20
Clinical T stage			
	T1	178	35.74
	T2	173	34.74
	T3	53	10.64
	T4	2	0.40
	NA	92	18.47
Lymph nodes			
	Positive	80	16.06
	Negative	327	65.66
	NA	91	18.27
PSA level			
	<10 ng/ml	425	85.34
	10–20 ng/ml	11	2.21
	>20 ng/ml	5	1.00
	NA	57	11.45

For all of the variables used in this study, the number and percentage of tumor patients with the characteristic was given in this table, except for *median and range was given for the patient age and the follow up time.

The Gleason score is a system of grading for prostate tissue that is based on how it looks under a microscope for describing how likely it is that a tumor will spread and how aggressive a prostate cancer tumor is in refs 51–53. The Gleason score is directly related to a number of clinical and histopathologic end points, including clinical stage, survival, progression to metastatic disease, tumor size, margin status, and pathologic stage. The Gleason score is often incorporated into nomograms used to predict the response to a specific therapy, such as surgery or radiotherapy, and also used as an important prognostic factor across all treatments for prostate adenocarcinoma in the present time. Gleason scores are always between 2 and 10; however, Gleason scores below 6 are not usually given because it is difficult to determine with certainty where the low-grade tumors are in fact cancerous. A high Gleason score indicates that the tumor is more likely to show aggressive behavior and therefore more likely to have spread outside of the gland to lymph nodes (metastases), and the cancer cells appear; a low Gleason score indicates that the tumor is less likely to show aggressive behavior and therefore less likely to have spread outside of the gland to lymph nodes (metastases), and the cancer cells appear more normal. In this study, to investigate the differences in Gleason score among patients in different k molecular subtypes, the Kruskal-Wallis test was performed. Among the tumors with a Gleason score, statistically significant associations between molecular subtypes classified by mutation profiles and Gleason score were observed by the Kruskal-Wallis test across multiple k’s (Table 2), demonstrating that mutation profiles were effective for classifying patients into clinically meaningful subtypes for prostate adenocarcinoma.

**Table 2:** The statistical association with molecular subtypes in different clinicopathologic characteristics across 498 tumor samples.

Parameter	2 subtypes	3 subtypes	4 subtypes	5 subtypes	6 subtypes	7 subtypes	8 subtypes	9 subtypes	
Gleason score	1.89E-02	1.07E-04	7.13E-09	5.91E-06	2.92E-07	4.84E-06	1.55E-06	8.97E-07	KW Test
Pathologic N stage	1.85E-01	3.61E-02	4.16E-04	1.96E-03	1.32E-03	3.93E-03	4.77E-04	1.04E-03	Chi-square
Pathologic T stage	1.77E-02	2.14E-02	2.60E-03	3.92E-02	2.47E-02	1.16E-02	1.14E-02	3.10E-02	Chi-square
Clinical T stage	4.00E-01	5.27E-01	1.26E-01	1.76E-01	4.59E-02	6.79E-02	2.32E-02	1.19E-01	Chi-square
Positive lymph nodes number	2.72E-01	3.85E-02	6.14E-05	3.38E-04	1.48E-04	1.05E-03	1.23E-04	3.59E-04	KW Test
PSA level	9.14E-01	9.77E-01	5.50E-01	5.57E-01	4.79E-01	7.60E-01	8.40E-01	2.56E-01	KW Test
Time to Death	9.79E-01	9.43E-01	5.65E-01	6.80E-01	6.15E-01	9.07E-01	7.38E-01	6.70E-01	Logrank test

(In this table, KW test indicated Kruskal-Wallis test).

Cancer staging is the process of determining how much cancer is in the body and where it is located^54, 55^. There are two types of cancer staging. The first is clinical staging, which is performed before treatment and determines how much cancer there is based on physical examination, imaging tests, and biopsies of affected areas. The second is pathologic staging, which is performed after treatment and can only be determined from individual patients who have had surgery to remove a tumor or explore the extent of the cancer. Because both clinical staging and surgical results are combined in pathologic staging, pathologic staging is more precise than clinical staging for measuring the extent of the cancer. In this study, except for the samples that were classified into 2 clusters, statistically significant associations were found between all of the different k molecular subtypes and pathologic N stage and pathologic T stage (Table 2). However, as shown in Table 2, no statistically significant associations were found between most the different k molecular subtypes and clinical T stage.

Lymph nodes are small, bean-shaped organs that act as filters along the lymph fluid channels. Cancer cells can spread to the lymph nodes from a cancer in any part of the body. When lymph nodes contain some cancer cells, they are called positive lymph nodes. If lymph nodes are free, or clear, of cancer, they are called negative lymph nodes^56^. The greater the number of positive lymph nodes, the more serious the cancer might be. Thus, the number of positive lymph nodes can be used by doctors to help determine the treatment plan for patients. In this study, to investigate differences in the number of positive lymph nodes between patients in different k clusters, Kruskal-Wallis tests were performed. There were statistically significant differences in the number of positive lymph nodes among the patients with subtypes 3–9. However, no significant difference in number of positive lymph nodes was observed between patients with subtype 2.

Prostate-specific antigen, or PSA, is a protein produced exclusively by cells of the prostate gland. The PSA test measures the level of PSA in a man’s blood, and this can help to detect prostate adenocarcinoma early before it grows and spreads outside the prostate^57^. It is normal for all men to have a small amount of PSA in their blood, and a raised PSA level may indicate prostate adenocarcinoma, an enlarged prostate, a noncancerous condition such as prostatitis, or simply aging. Conversely, low levels of PSA do not rule out the possibility of prostate adenocarcinoma. To investigate the differences in PSA level among patients in different k molecular subtypes, statistical analysis was performed. The statistical analysis using nonparametric Kruskal-Wallis tests failed to demonstrate a significant association between the subtypes and the PSA level (Table 2), suggesting that this feature is independent of the molecular subtype.

### Survival of prostate adenocarcinoma patients with respect to subtypes

**Figure 3:**
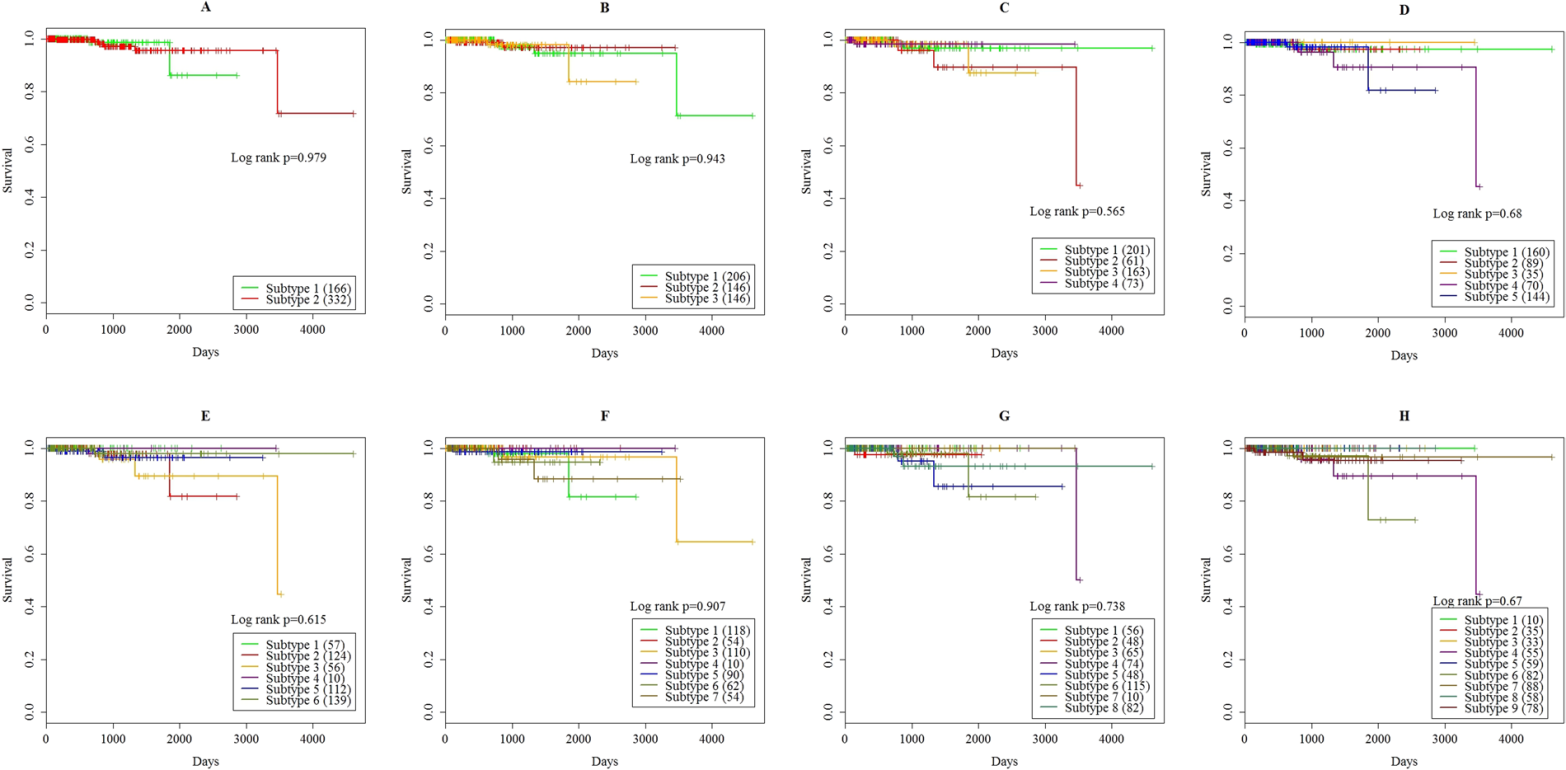
Survival curves of 498 tumor patients with respect to the (**A**) 2 subtypes, (**B**) 3 subtypes, (**C**) 4 subtypes, (**D**) 5 subtypes, (**E**) 6 subtypes, (**F**) 7 subtypes, (**F**) 8 subtypes, (**G**) 9 subtypes. The numbers in the brackets indicated the corresponded number of samples for this subtype. As shown in this figure, no statistically significant difference was found by the log-rank tests. This is because at this time, the follow-up time of this cohort remains very limited, when additional follow-up data are available in the future time, it is possible that the differences will become apparent.

Next, we investigated the association between subtypes and survival. The median patient ages and follow-up times are shown in Table 1. As shown in this table, the median overall patient age was 61 years (range, 41–78 years), the median overall follow-up time was 512 days (range: 1 to 4602 days), and only 8 patients died of prostate adenocarcinoma. To further investigate the clinical relevance of the molecular subtypes, a Kaplan-Meier Survival analysis on the 498 samples was performed with the package survival (version 2.39-5) in R. A log-rank test was used to assess significance. As shown in Fig. 3 and Table 2, the survival curves were not statistically significantly different between the molecular subtypes for all cases. Prostate adenocarcinoma has one of the highest survival rates of any type of cancer. Most of the patients who diagnosed with prostate adenocarcinoma had a high chance of living for at least five more years. Ninety-eight percent of men who diagnosed with prostate adenocarcinoma were alive after 10 years, and 95 percent lived for at least 15 years^58, 59^. At the time of this analysis, the follow-up time of this cohort remains very limited; thus, the survival analyses was quite exploratory due to the low mortality rates for this cohort, and no significant difference among the molecular subtypes in the survival curves was expected. When additional follow-up data are available in the future, it is possible that the differences will become apparent.

### Robustness of the subtyping in prostate adenocarcinoma

**Figure 4:**
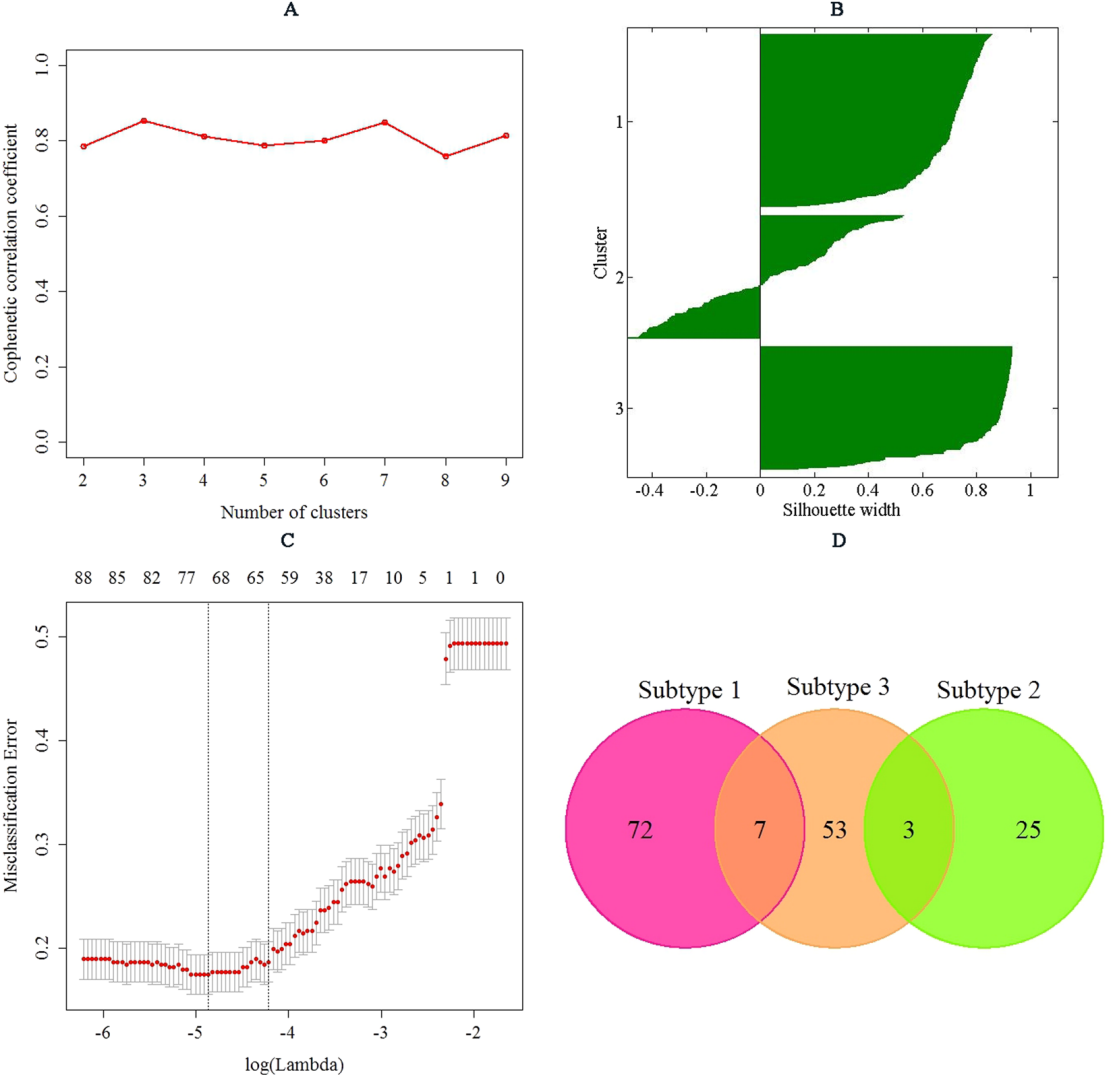
(**A**) Cophenetic correlation coefficient for clusters k = 2 to k = 9. The figure demonstrates that maximum cophenetic correlation coefficient occurred for cluster k = 3. (**B**) Silhouette plot for the robust cluster. (**C**) The misclassification error rates in the jackknife test. Each dot represents a lambda value along the path, with error bars to give a confidence interval for the misclassification error rate. This figure illustrated that the highest accuracy (overall misclassification error) was 82.54% by the jackknife test. (**D**) Venn diagram of different biomarkers between three molecular subtypes. This figure illustrated that among 170 unique biomarker genes, 7 biomarker genes were in common between subtype 1 and subtype 3, and 3 biomarker genes were in common between subtype 2 and subtype 3.

**Figure 5:**
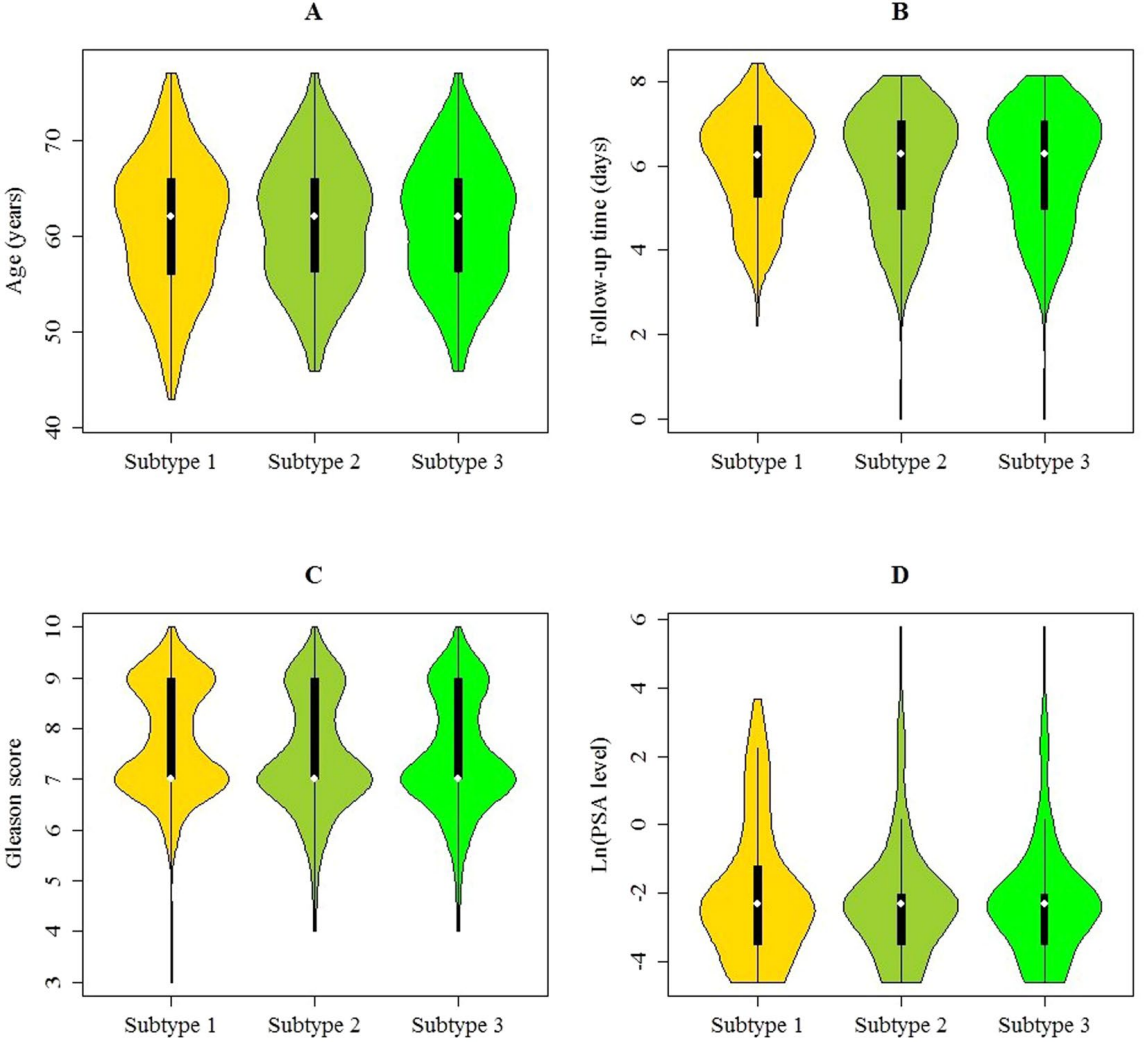
The violin plots of (**A**) patient age, (**B**) follow-up time, (**C**) Gleason score, and (**D**) PSA level for the robust clustering.

In this study, we computed the clustering for k = 2 to k = 9 and used the cophenetic correlation coefficient to determine the cluster that yielded the most robust clustering^35^. The cophenetic correlation coefficient was computed based on the consensus matrix of the GNMF clustering, and it measured how reliably the same samples were assigned to the same cluster across many iterations of the clustering algorithm with random initializations. The cophenetic correlation coefficient lies between 0 and 1, with higher values indicating more stable cluster assignments. Based on the largest observed correlation coefficient for all tested values of k (Fig. 4A), the number of clusters k = 3 was selected as the most robust consensus GNMF clustering of 498 samples using the 500 most variable genes. As such, the unsupervised GNMF consensus analysis of the mutation profile data from 498 tumor samples revealed three clusters of samples: subtype 1 (n = 206, 41.37%), subtype 2 (n = 146, 29.32%), and subtype 3 (n = 146, 29.32%). The median patient ages were 62 years (range: 43–77 years), 62 years (range: 46–77 years), 60.5 years (range: 43–78 years) for subtype 1, subtype 2, and subtype 3, respectively (Fig. 5A). According to the Kruskal-Wallis test, no association was detected between the molecular subtypes and the patient ages (P-value = 5.67E-1), indicating that patient ages were independent of the molecular subtype. The median follow-up times were 530.50 days (range: 9 to 4604 days), 534.50 days (range: 1 to 3447 days), and 442.50 days (range: 1 to 2859 days) for subtype 1, subtype 2, and subtype 3, respectively (Fig. 5B). No significant differences were detected between the three molecular subtypes by Kaplan-Meier analysis (P-value = 9.43E-1, Logrank test). In addition, we found that tumors classified as subtype 1 had significantly higher Gleason scores (mean Gleason score = 7.77) than the tumors classified as subtype 2 (mean Gleason score = 7.49, P-value = 1.74E-02, Wilcoxon rank sum test) and the tumors classified as subtype 3 (mean Gleason score = 7.30, P-value = 2.19E-05, Wilcoxon rank sum test) (Fig. 5C). These three molecular subtypes were further investigated using a Chi-square test to explore the differences of the subtypes in pathologic N stage characteristics. As observed in Table 3, the patients in subtype 3 displayed low enrichment in the pathologic N1 stage (8.90%, n = 13), whereas those in subtype 1 and the subtype 2 displayed higher enrichment in the pathologic N1 stage (19.42%, n = 40 for the subtype 1 and 17.81%, n = 26 for the subtype 2, respectively) than the patients in subtype 3, and the differences between them were significant according to the Chi-square test (P-value = 2.29E-2 for subtype 1 versus subtype 3, and P-value = 3.58E-2 for subtype 2 versus subtype 3). According to the pathologic T stage, the percentages for the subtype 3 were 47.26% for the pathologic T2 stage (n = 69) and 50.00% for the pathologic T3 stage (n = 73) (Table 3). For subtype 1 and subtype 2, the percentages of patients with a pathologic T3 stage were 63.11% (n = 130) and 61.64% (n = 90), respectively, indicating more patients with a higher pathologic stage in these two subtypes. Additionally, statistically significant differences in pathologic T stage characteristics were observed between subtype 1 and subtype 3 (P-value = 1.35E-2, Chi-square test), and subtype 2 and subtype 3 (P-value = 3.86E-2, Chi-square test). In this study, among 498 patients, 80 patients had positive lymph nodes and 327 patients had negative lymph nodes. There was a significant difference in the robust subtypes between patients with positive lymph nodes and patients with negative lymph nodes (P-value = 4.69E-2, Chi-square test). The average PSA levels for the three subtypes were 1.42 ng/mL, 3.19 ng/mL, and 0.74 ng/mL, respectively (Table 3 and Fig. 5D). Therefore, compared with those in subtype 1 and subtype 2, subtype 3 was associated with the lowest PSA level (P-value = 9.61E-1 and P-value = 9.14E-1 versus subtype 1 and subtype 2, respectively; Wilcoxon test).

**Table 3:** Clinicopathological characteristics for each molecular subtype in the robust clustering.

Parameter		Subtype 1	Subtype 2	Subtype 3
Patient number		206	146	146
Patient age (yrs)				
	Median	62	62	60.5
	Range	43–77	46–77	41–78
Follow-up time (days)				
	Median	530.50	534.50	442.50
	Range	9–4604	1–3447	1–2859
Gleason score				
	Mean*	7.77	7.49	7.30
	2–6	13	19	21
	7	92	70	84
	8–10	101	57	41
Pathologic N stage				
	N0	145	94	107
	N1	40	26	13
	NA	21	26	26
Pathologic T stage				
	T2	69	50	69
	T3	130	90	73
	T4	5	2	4
	NA	2	4	0
Clinical T stage				
	T1	71	52	55
	T2	68	47	58
	T3	25	17	11
	T4	0	0	2
	NA	42	30	20
Lymph nodes				
	Positive	41	26	13
	Negative	138	91	98
	NA	27	29	35
PSA level				
	Mean*	1.42 ng/mL	3.19 ng/mL	0.74 ng/mL
	<10 ng/ml	172	126	127
	10–20 ng/ml	7	2	2
	>20 ng/ml	2	2	1
	NA	25	16	16

(*In this table, all of the median values for three molecular subtypes were same, so the mean values were used to replace the median values).

The Elastic Net predictor approach was used to classify samples based on the smoothed mutation profile. To choose important features to discriminate three types of prostate adenocarcinomas, the top 500 genes with the highest variance were selected to build the classifier. Silhouette width was also computed to identify samples with strong membership to their assigned subtype (Supplementary Table S2 and Fig. 4B). Finally, 401 samples with silhouette width >0.20 were retained as the core samples to build our classifier (Supplementary Table S3). A jackknife test was used to optimize the Elastic Net mixing parameter: alpha, and the Elastic Net regularization parameter: lambda. The accuracy was calculated to assess the ability of the Elastic Net to separate one subtype from the others. The Elastic Net predictor approach obtained the highest accuracy (overall misclassification error) of 82.54% in assigning the molecular subtypes (Fig. 4C). In addition, 401 core samples were used to select differential biomarker genes for each subtype using the Elastic Net algorithm. The biomarker genes were selected according to their correlation with the group assignment of subtype 1, subtype 2, and subtype 3.

With a 82.54% accuracy in assigning the molecular subtypes by the jackknife test, we identified 79 biomarker genes, 28 biomarker genes, and 63 biomarker genes corresponding to three subtypes with non-zero coefficients. This set of biomarker genes was able to assign prostate adenocarcinoma to one of three subtypes. The results of the biomarker genes selected by the Elastic Net algorithm for each subtype are represented in Supplementary Table S4. Figure 4D illustrates the pairwise comparisons of three biomarker genes lists that were different among the subtypes of prostate adenocarcinoma. As shown in this Venn diagram, among 170 unique biomarker genes, 7 biomarker genes were in common between subtype 1 and subtype 3, and 3 biomarker genes were in common between subtype 2 and subtype 3.

All of the biomarker genes that correlated with each subtype were input into the online software DAVID. The threshold used in this study was P-value < 0.05 after the Benjamini correction. The resulting KEGG pathway enrichment terms and GO enrichment terms are summarized in Supplementary Tables S5 and S6. The 79 biomarker genes of subtype 1 were enriched with 28 KEGG pathways, 2 molecular functions, 167 biological processes and 3 cellular components; the 28 biomarker genes of subtype 2 were enriched with 1 KEGG pathway, 1 molecular function, 9 biological processes and 1 cellular component; the 63 biomarker genes of subtype 3 were enriched with 5 KEGG pathways, 1 molecular function, 8 biological processes and 3 cellular components. Of the biomarker genes, approximately 30 (37.97%) for subtype 1, 14 (50.00%) for subtype 2, and 26 (41.27%) for subtype 3 were enriched in olfactory transduction (KEGG ID: 04740). In addition, we observed that all 5 KEGG pathways that were enriched by 63 biomarker genes of subtype 3 overlapped with those of subtype 1 however, only 7 biomarker genes were overlapped by these two subtypes. The KEGG pathway enrichment of these biomarker genes also indicated that they were likely to be enriched in cancer-related pathways such as pathways in cancer (KEGG ID: 05200), pancreatic cancer (KEGG ID: 05212), prostate cancer (KEGG ID: 05215), bladder cancer (KEGG ID: 05219), small cell lung cancer (KEGG ID: 05222), non-small cell lung cancer (KEGG ID: 05223), and so on. We also performed a GO enrichment analysis on these biomarker genes, and the results suggested that all classes of biomarker genes were likely to be enriched in the sensory perception of smell (GO:0007608), olfactory receptor activity (GO:0004984), cell surface receptor linked signal transduction (GO:0007166), plasma membranes (GO:0005886), and so on (Supplementary Table S6).

## Discussion

In this study, by using the molecular network and the somatic mutation profiles, the 498 prostate adenocarcinoma samples were stratified into different molecular subtypes that were both biologically and clinically informative. As we know, this is the first time that the prostate adenocarcinoma patients were stratified by the somatic mutation profiles. Unlike expression and other omics profiles, the somatic mutations were only the differential measurements between tumor and normal tissue, so, a quantitative value could not be assigned for each patient. Thus, using the mutation profiles to classify the prostate adenocarcinoma samples may be very challenging. Here, in this study, we not only deal with the sparsity of the somatic mutation profiles of cancers but also providing some biologically and clinically meaningful knowledge for effective subtyping of cancers. The work presented in this study may enable the therapy and prognostication of prostate adenocarcinoma more feasible in clinical research, and these findings may contribute to better elucidate the performance of clinical outcome as well. Benefit from the meaningful results of subtyping prostate adenocarcinoma, we will strive to use the somatic mutation data in other cancers for classifying patients into distinct molecular subtypes in our future work.

Unlike the expression or methylation data of tumors where almost all genes are assigned quantitative values for each patient, the somatic mutation profiles are extremely sparse. Hence, it would be very challenge to use the sparsity of mutation data to stratify patients into subtypes that are both biologically and clinically informative. In this study, to overcome the challenge of the sparsity of mutation data, the network propagation method was used to spread the influence of each mutation in the protein interaction network, and smoothed mutation profiles of prostate adenocarcinoma samples were generated. The GNMF algorithm was applied to stratify the smoothed mutation profiles of prostate adenocarcinoma samples into different molecular subtypes without applying any biological or clinical information. Importantly, these subtypes derived by mutation profiles were associated with a significant difference in clinicobiological characteristics, indicating that prostate tumors can at least be usefully classified according to their mutation profile patterns, and these tumor subtypes may provide some help for improving treatment stratification and prognostication of prostate adenocarcinoma. In addition, we identified three robust molecular subtypes of prostate adenocarcinoma individualized by distinct clinicobiological characteristics; the biomarkers for each subtype were selected by the Elastic Net predictor approach with an overall accuracy of 82.54%. To the best of our knowledge, this is the first time that a network-based approach integrating the somatic mutation profiles and protein interaction network has been used to stratify prostate adenocarcinoma in an unsupervised fashion. Based on these meaningful and effective stratification results of prostate adenocarcinoma, we can conclude that the protein interaction networks used in this study can address the sparsity of somatic mutation data. We will strive to use our stratification method for other somatic mutation profiles and in other cancer subtyping area which need further exploration in our future work.

Although our study has some advantages in stratifying prostate adenocarcinoma, there were several limitations in our study. First, until now, there has been no gold standard for evaluating the performance of the molecular subtypes of prostate adenocarcinoma. The cophenetic correlation coefficient used in this study can only evaluate the stable cluster assignments for each subtype, but validating subtype classification was difficult because the true subtypes for each sample were still not known until now. Thus, it is difficult to assess which stratification is more meaningful and more accurate. Second, many types of protein-protein interactions and protein interaction networks have been published for humans in recent years; however, the optimal types of interactions and optimal networks for stratifying tumors of prostate adenocarcinoma into truly molecular subtypes that are biologically informative and have associations with clinical outcomes are still unclear. Third, the performance of our method depended not only on network smoothing but also on the GNMF algorithm; hence, various tuning parameters, such as r in the network propagation step and the lambda in the GNMF algorithm, were involved in our method. The effect of r and lambda on the performance of our method and how to obtain the optimal value of these two values to stratify tumors of prostate into truly molecular subtypes are still unclear.

**Figure 6:**
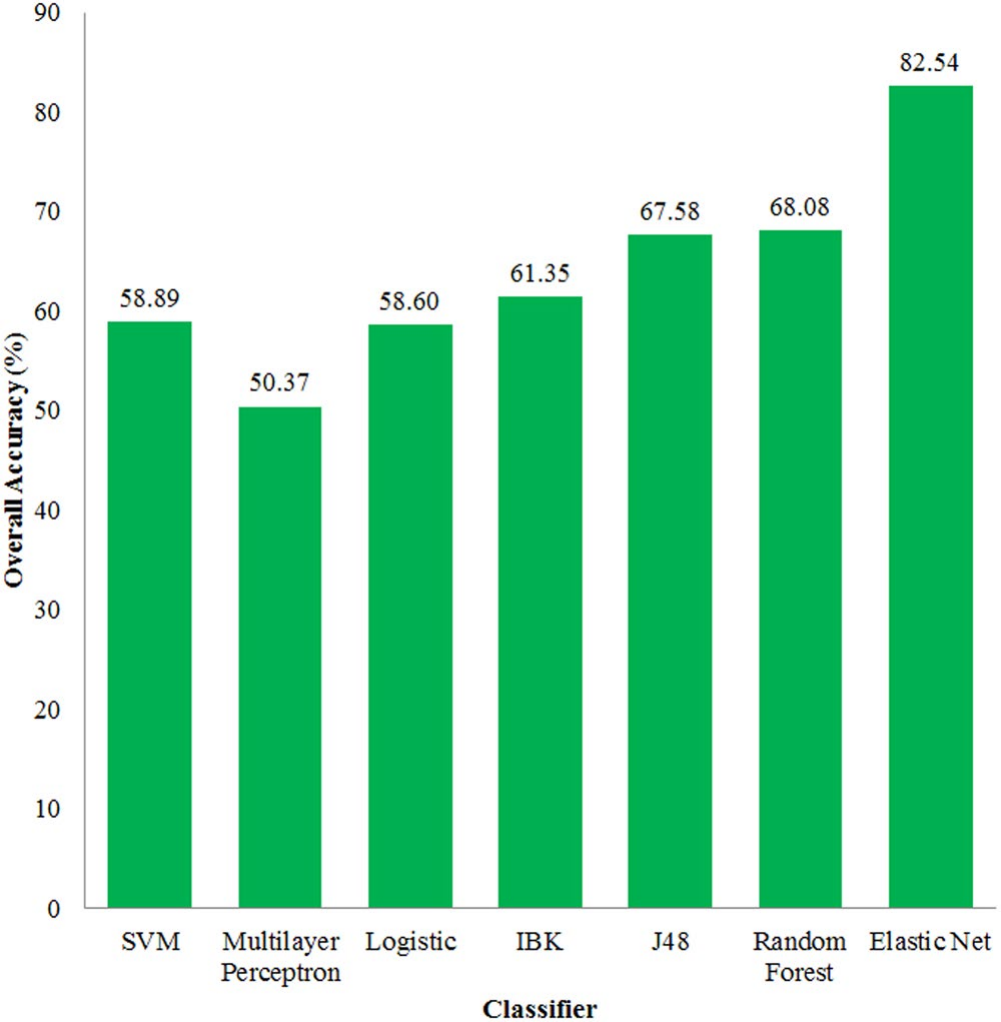
The comparison results of the Elastic Net with other classifiers.

The support vector machine (SVM), multilayer perceptron, logistic, IBK, J48 and random forest algorithms that were implemented in Weka (version 3.8.0) were used to compare the predictive results with the Elastic Net. Based on the top 500 genes with the highest variance, 401 core samples were predicted by these classifiers. The jackknife test was used to evaluate the performance of these classifiers, and the overall accuracies were illustrated in Fig. 6. From this figure, we can see that the overall accuracy of the Elastic Net was 82.54%, which were higher than those of the other classifiers. The successful prediction clearly indicated that the Elastic Net was a promising approach.

To compare the classification accuracy of the different number of the top selected genes, eight benchmark datasets were constructed. The detailed number of the top selected genes for each benchmark dataset was shown in Table 4. The performance was compared based on eight different benchmark datasets, and the predictive results obtained by the jackknife tests were shown in Table 4. From this table, we can see that the classification accuracy of our classifier was affected by the numbers of top selected genes, and the highest overall accuracy of 82.54% was achieved when the top 500 genes was used as the parameter of our classifier. So, the prediction results in Table 4 can indicate that the top 500 genes were suitable to be selected as the input parameters of Elastic Net.

**Table 4:** The predictive results of eight benchmark datasets.

Dataset	Number of genes	Acc (%)
Benchmark dataset 1	300	69.58
Benchmark dataset 2	400	73.32
Benchmark dataset 3	500	82.54
Benchmark dataset 4	600	80.05
Benchmark dataset 5	700	79.55
Benchmark dataset 6	800	77.81
Benchmark dataset 7	900	77.81
Benchmark dataset 8	1000	75.06

One goal of our study was to find a set of genes that can accurately classify tumor samples; thus, our study also built a classifier that identifies the three distinct molecular subtypes of prostate adenocarcinoma with an accuracy of 82.54% based on 170 genes that correspond to three distinct molecular subtypes. The online enrichment analysis tool DAVID was used to identify enriched biological functions for each of the subtype genes. Within these genes, enriched KEGG pathways were related to a large cluster of genes relating to cancer, such as pathways in cancer, pancreatic cancer, prostate cancer, bladder cancer, small cell lung cancer, and non-small cell lung cancer; and enriched GO terms were related to clusters of genes governing the sensory perception of smell, olfactory receptor activity, cell surface receptor linked signal transduction, plasma membranes, and so on. Our method provided an accurate and rapid assay for identifying these molecular subtypes of prostate adenocarcinoma. The efficiency of our method in finding a number of predictive biomarker genes can facilitate the search for new diagnostic tools in prostate adenocarcinoma and the selection of drug targets of gene therapy for prostate adenocarcinoma. As the molecular subtypes become part of risk stratification in cancer research, it may be used to identify genes that are predictive for response to chemotherapy. Our findings may provide some useful help for the discovery of new biomarkers and personalization of prostate adenocarcinoma care. Future work will focus on validating the predictive capacity of these identified biomarkers and the functional elucidation of these identified biomarkers. Recently, some powerful DNA/RNA sequence analysis tools^60–65^ developed based on the concept of PseAAC^66^ addressing the current topic. In addition, as shown in a series of recent publications^38, 41–46, 49, 67–76^ in demonstrating new prediction/classification methods, user-friendly and publicly accessible web-servers will significantly enhance their impacts^77^; we shall make efforts in our future work to provide a web-server for the method reported in this paper.

## Methods

### Dataset

The level 2 somatic mutation data sets for prostate adenocarcinoma were downloaded from The Cancer Genome Atlas (TCGA) data portal (https://tcga-data.nci.nih.gov/tcga/) on 5 February 2016. The number of prostate adenocarcinoma samples was 498, and mutations occurred in 14268 genes. The clinical variables for prostate adenocarcinoma were also obtained from the TCGA data portal on 5 February 2016.

### Protein-protein interaction data

The human protein-protein interaction data were downloaded from the Search Tool for the Retrieval of Interacting Genes (STRING) database (version 9) (http://string-db.org/)^78^. The STRING database provides comprehensive coverage on both experimental evidence for protein-protein interactions as well as interaction information predicted by comparative genomics and text mining. An advantage of the STRING database is that a scoring scheme is provided for every single interaction to reflect the evidence of interactions. In this study, a high-confidence human PPI network was constructed using the interactions with a score >0.7 in the STRING database, and 428,238 interactions among 13,962 proteins were included in this network.

### Random walk with restart algorithm (RWRA)

In this study, the random walk with restart (RWRA)^79^ as implemented in the R package dnet (version 1.0.9) was used as a variant of the random walk. In a given graph, the random walk with restart mimics a walker who moves from its current node to a randomly selected neighbor or goes back to the source node with a probability r. Formally, the random walk with restart is described as follows:

1 $${p}^{t+1}=(1-r)W{p}^{t}+r{p}^{0}$$

where W is the column-normalized adjacency matrix of the graph, p^t^ is a vector holding the scores of the nodes at time step t, p^t+1^ is a vector holding the scores of the nodes at time step t + 1, and r is the restart probability ranging from 0 to 1. In addition, p^0^ is the initial probability vector that the equal probabilities are assigned to each source node in the graph. All nodes are ranked according to the steady state probability vector p^∞^. This is numerically obtained by repeating the iterations until the change between p^t^ and p^t+1^ is smaller than 10^−6^.

### Graph-regularized Nonnegative Matrix Factorization (GNMF)

Non-negative Matrix Factorization (NMF) is an unsupervised, parts-based learning algorithm that has been applied on the analysis of data matrices whose elements are nonnegative^34, 80^. However, the NMF performs the learning in a Euclidean space; thus, it usually fails to discover the intrinsic discrimination and geometrical structure of the data space, which is very important to real-world applications. To avoid this problem, the Graph-regularized Non-negative Matrix Factorization (GNMF) algorithm is introduced by incorporating a geometrically based regularizer^34^. Similar to the NMF, the GNMF algorithm iteratively computes an approximation *A* ~ *WH* by minimizing the objective function as follows:

2 $${\begin{array}{cc}\mathop{Min}\limits_{W,H > 0} & \Vert A-W{H}^{T}\Vert \end{array}}^{2}+\lambda Tr({H}^{T}LH)$$

where A is a positive matrix of size *n* × *m*; W and H are the *n* × *r* and *r* × *m* non-negative matrices, respectively. The regularization parameter *λ* ≥ 0 controls the smoothness of the new representation, *Tr*(·) denotes the trace of a matrix, and L is called the Laplacian graph.

### Elastic Net analysis

The Elastic Net predictor approach as implemented in the R package glmnet (version 2.0–5) was used to predict the molecular subtype of a given sample^36^. The jackknife test was used to optimize algorithm-specific parameters, including the Elastic Net mixing parameter: alpha, and the regularization parameter: lambda. Next, the Elastic Net algorithm was also used to select distinctive features for each molecular subtype. The features identified within each subtype were considered as the biomarkers of this subtype.

### Enrichment analysis

The biomarker genes of each subtype were input into the functional annotation tool DAVID (http://david.abcc.ncifcrf.gov/home.jsp) for a corresponding GO and KEGG pathway enrichment analysis^37^. The enrichment p-value was determined by hypergeometric tests and then corrected by the Benjamin multiple testing correction method.

### Statistical analysis

Associations between and among clinical and molecular subtypes were evaluated by a Chi-square test (categorical versus categorical), Wilcoxon rank sum test, or Kruskal-Wallis test (continuous versus continuous) according to the nature of the data levels for each pair. Differences between survival curves were tested using the two-sided Log-rank test as implemented in the R package survival (version 2.39-5). The freely available R software and a significance level of P-value < 0.05 (two-tailed probability) were used for all statistical tests. In addition, the jackknife test^81–84^ was used to examine the prediction power of the Elastic Net algorithm.

## Electronic supplementary material

Supplementary tabels
