# Supplementary material for: Molecular classification of prostate adenocarcinoma by the integrated somatic mutation profiles and molecular network
Source: Sci Rep. 2017 Apr 7;7:738. doi: 10.1038/s41598-017-00872-8 (PMC5429686; doi:10.1038/s41598-017-00872-8)
Supplement: Supplementary file 1 — Supplementary tabels [file 41598_2017_872_MOESM1_ESM.pdf]

Molecular classification of prostate adenocarcinoma by the integrated somatic mutation profiles and molecular network

Lei Yang[a]*†, Shiyuan Wang[a]†, Meng Zhou[a], Xiaowen Chen[a], Wei Jiang[a], Yongchun Zuo[b]*, Yingli Lv[a]*

[a]College of Bioinformatics Science and Technology, Harbin Medical University, Harbin 150081, China

[b]The Key Laboratory of Mammalian Reproductive Biology and Biotechnology of the Ministry of Education, Inner Mongolia University, Hohhot 010021, China

†Equal contributors

*To whom correspondence should be addressed

Lei Yang, Tel: +86 451 8666 9617; Fax: +86 451 8666 9617; Email address: yanglei_hmu@163.com

Yongchun Zuo, Tel: +86 471 5227683; Fax: +86 471 5227683; Email address: yczuo@imu.edu.cn

Yingli Lv, Tel: +86 451 8666 9617; Fax: +86 451 8666 9617; Email address: lvyingli_hmu@163.com

Table S1 List of samples belonging to each cluster in different k clusters.

Sample Name	K=2	K=3	K=4	K=5	K=6	K=7	K=8	K=9
TCGA-2A-A8VL-01	2	2	1	1	5	5	4	7
TCGA-2A-A8VO-01	2	2	4	1	5	5	2	9
TCGA-2A-A8VT-01	1	1	2	4	3	7	5	4
TCGA-2A-A8VV-01	2	1	1	1	6	3	8	7
TCGA-2A-A8VX-01	2	1	1	2	6	6	3	7
TCGA-2A-A8W1-01	2	1	1	2	6	6	3	5
TCGA-2A-A8W3-01	1	3	3	5	2	1	6	8
TCGA-2A-AAYF-01	2	2	4	1	5	5	2	9
TCGA-2A-AAYO-01	1	3	3	5	2	1	6	6
TCGA-2A-AAYU-01	1	3	3	5	2	1	6	8
TCGA-4L-AA1F-01	2	2	1	1	5	5	4	9
TCGA-CH-5737-01	2	2	1	1	6	3	8	7
TCGA-CH-5738-01	2	2	4	3	5	5	2	3
TCGA-CH-5739-01	1	3	3	5	1	2	1	8
TCGA-CH-5740-01	2	2	4	1	5	5	2	9
TCGA-CH-5741-01	2	1	1	1	6	3	4	7
TCGA-CH-5743-01	2	1	1	1	6	3	8	5
TCGA-CH-5744-01	1	3	3	5	2	1	6	6
TCGA-CH-5745-01	2	1	1	1	6	6	3	5
TCGA-CH-5746-01	1	3	3	5	2	1	6	6
TCGA-CH-5748-01	2	2	1	1	5	5	4	3
TCGA-CH-5750-01	2	1	1	2	6	6	3	5
TCGA-CH-5751-01	2	1	1	1	6	3	8	7
TCGA-CH-5752-01	2	1	1	1	6	3	8	7
TCGA-CH-5753-01	1	3	3	5	1	2	1	8
TCGA-CH-5754-01	2	1	1	2	1	6	3	5
TCGA-CH-5761-01	2	1	2	4	3	7	5	4
TCGA-CH-5762-01	2	1	1	1	5	5	4	9
TCGA-CH-5763-01	1	3	3	5	2	1	6	8
TCGA-CH-5764-01	2	1	1	2	6	6	3	5
TCGA-CH-5765-01	2	2	4	3	5	5	4	3
TCGA-CH-5766-01	1	3	3	5	1	2	1	8
TCGA-CH-5767-01	1	3	3	5	2	1	6	8
TCGA-CH-5768-01	2	1	1	2	6	6	3	5
TCGA-CH-5769-01	2	2	4	1	5	5	2	9
TCGA-CH-5771-01	2	2	2	4	3	7	5	4
TCGA-CH-5772-01	2	1	1	2	6	6	8	5
TCGA-CH-5788-01	2	1	1	1	6	3	4	7
TCGA-CH-5789-01	2	2	4	3	5	5	4	3

TCGA-CH-5790-01	1	3	3	5	2	1	6	6
TCGA-CH-5791-01	2	2	4	1	5	5	2	9
TCGA-CH-5792-01	2	1	1	2	6	3	3	5
TCGA-CH-5794-01	2	2	4	3	5	5	2	3
TCGA-EJ-5494-01	2	1	1	1	6	3	8	7
TCGA-EJ-5495-01	2	2	1	1	6	3	8	3
TCGA-EJ-5496-01	2	2	4	1	5	5	2	9
TCGA-EJ-5497-01	1	3	3	5	2	1	6	8
TCGA-EJ-5498-01	2	1	1	1	6	3	8	7
TCGA-EJ-5499-01	2	1	1	2	6	6	3	5
TCGA-EJ-5501-01	2	2	1	3	6	3	3	5
TCGA-EJ-5502-01	1	3	3	5	2	1	6	6
TCGA-EJ-5503-01	2	2	4	3	6	6	3	5
TCGA-EJ-5504-01	2	1	1	1	6	3	8	7
TCGA-EJ-5505-01	2	2	4	3	6	6	3	5
TCGA-EJ-5506-01	2	2	1	1	5	5	4	9
TCGA-EJ-5507-01	2	1	2	4	3	7	5	4
TCGA-EJ-5508-01	2	2	4	1	5	7	2	9
TCGA-EJ-5509-01	2	1	1	1	5	5	4	9
TCGA-EJ-5510-01	1	3	3	5	2	2	1	8
TCGA-EJ-5511-01	2	2	1	1	5	3	4	7
TCGA-EJ-5512-01	2	1	1	1	6	3	8	3
TCGA-EJ-5514-01	2	1	2	4	3	7	5	4
TCGA-EJ-5515-01	1	3	3	5	2	1	6	6
TCGA-EJ-5516-01	1	3	3	5	2	2	1	8
TCGA-EJ-5517-01	1	3	3	5	2	1	6	6
TCGA-EJ-5518-01	2	2	1	1	5	3	8	7
TCGA-EJ-5519-01	2	2	4	3	5	5	4	3
TCGA-EJ-5521-01	2	1	2	4	3	7	5	4
TCGA-EJ-5522-01	2	2	1	1	5	5	4	9
TCGA-EJ-5524-01	1	3	3	5	2	1	6	6
TCGA-EJ-5525-01	2	1	2	4	3	3	4	4
TCGA-EJ-5526-01	2	1	1	1	6	3	8	7
TCGA-EJ-5527-01	2	2	1	1	5	5	4	3
TCGA-EJ-5530-01	2	1	1	1	6	3	8	7
TCGA-EJ-5531-01	2	1	1	2	1	6	3	5
TCGA-EJ-5532-01	1	3	3	5	2	1	6	6
TCGA-EJ-5542-01	1	3	3	5	1	2	1	8
TCGA-EJ-7115-01	2	1	1	1	6	3	8	7
TCGA-EJ-7123-01	2	1	1	2	6	6	3	5
TCGA-EJ-7125-01	1	3	3	5	2	1	6	8

TCGA-EJ-7218-01	2	2	1	1	6	3	8	7
TCGA-EJ-7312-01	1	3	3	5	2	1	6	6
TCGA-EJ-7314-01	1	3	3	5	2	1	6	8
TCGA-EJ-7315-01	2	1	2	4	3	7	5	4
TCGA-EJ-7317-01	1	3	3	5	1	2	1	8
TCGA-EJ-7318-01	2	1	1	1	6	3	8	7
TCGA-EJ-7321-01	2	1	1	1	6	3	8	7
TCGA-EJ-7325-01	2	2	4	1	5	5	2	9
TCGA-EJ-7327-01	1	3	3	5	2	1	6	8
TCGA-EJ-7328-01	2	1	1	2	1	6	3	2
TCGA-EJ-7330-01	1	3	3	5	2	1	6	8
TCGA-EJ-7331-01	2	1	1	1	6	3	8	7
TCGA-EJ-7781-01	2	1	2	4	6	3	4	7
TCGA-EJ-7782-01	1	3	3	5	2	1	6	8
TCGA-EJ-7783-01	2	2	1	1	6	3	4	7
TCGA-EJ-7784-01	2	1	1	1	6	3	8	7
TCGA-EJ-7785-01	2	1	1	2	6	6	3	5
TCGA-EJ-7786-01	2	2	4	1	5	5	2	7
TCGA-EJ-7788-01	1	3	3	5	2	1	6	6
TCGA-EJ-7789-01	2	1	1	2	6	6	3	5
TCGA-EJ-7791-01	2	2	4	1	5	5	2	9
TCGA-EJ-7792-01	1	3	3	5	2	1	6	6
TCGA-EJ-7793-01	2	1	1	1	6	3	8	7
TCGA-EJ-7794-01	1	3	3	5	2	1	6	6
TCGA-EJ-7797-01	2	1	1	2	6	6	8	5
TCGA-EJ-8468-01	1	3	3	2	1	2	1	2
TCGA-EJ-8469-01	2	1	1	1	6	3	8	7
TCGA-EJ-8470-01	1	3	3	5	2	1	6	6
TCGA-EJ-8472-01	2	1	2	4	3	7	5	4
TCGA-EJ-8474-01	2	1	1	2	6	6	3	5
TCGA-EJ-A46B-01	1	3	3	2	1	2	1	2
TCGA-EJ-A46D-01	1	3	3	5	2	1	6	6
TCGA-EJ-A46E-01	2	1	3	5	1	2	1	2
TCGA-EJ-A46F-01	2	1	3	5	1	2	1	2
TCGA-EJ-A46G-01	2	2	1	3	5	3	8	3
TCGA-EJ-A46H-01	2	2	1	1	5	5	4	9
TCGA-EJ-A46I-01	1	3	3	5	2	1	6	6
TCGA-EJ-A65B-01	2	2	4	1	5	5	2	7
TCGA-EJ-A65D-01	2	1	1	2	6	6	3	5
TCGA-EJ-A65E-01	1	1	3	2	1	2	1	2
TCGA-EJ-A65F-01	2	2	1	3	6	3	3	5

TCGA-EJ-A65G-01	1	3	3	5	2	1	6	6
TCGA-EJ-A65J-01	1	3	3	5	2	1	6	6
TCGA-EJ-A65M-01	1	3	3	5	2	1	6	8
TCGA-EJ-A6RA-01	1	3	3	5	2	1	6	6
TCGA-EJ-A6RC-01	2	2	4	3	5	5	2	3
TCGA-EJ-A7NF-01	2	2	1	4	5	5	4	9
TCGA-EJ-A7NG-01	1	3	3	5	2	1	6	6
TCGA-EJ-A7NH-01	2	1	2	4	3	7	5	4
TCGA-EJ-A7NJ-01	2	1	1	2	1	2	3	2
TCGA-EJ-A7NK-01	1	3	3	5	2	1	6	6
TCGA-EJ-A7NM-01	2	2	1	4	5	5	4	9
TCGA-EJ-A7NN-01	2	1	3	2	1	2	1	2
TCGA-EJ-A8FN-01	2	2	4	3	4	4	7	1
TCGA-EJ-A8FO-01	1	3	3	5	2	1	6	6
TCGA-EJ-A8FP-01	1	3	3	5	2	1	6	6
TCGA-EJ-A8FS-01	2	2	1	1	5	5	4	9
TCGA-EJ-A8FU-01	2	2	4	3	6	6	3	5
TCGA-EJ-AB20-01	1	3	3	5	2	1	6	6
TCGA-EJ-AB27-01	1	1	1	4	6	6	8	7
TCGA-FC-7708-01	1	3	3	2	1	2	1	2
TCGA-FC-7961-01	2	2	4	1	5	5	2	9
TCGA-FC-A4JI-01	1	3	3	5	2	1	6	8
TCGA-FC-A5OB-01	2	1	1	2	6	6	3	5
TCGA-FC-A66V-01	2	1	1	2	6	6	8	5
TCGA-FC-A6HD-01	2	1	1	2	6	6	3	5
TCGA-FC-A8O0-01	2	1	1	2	6	6	3	5
TCGA-G9-6329-01	2	1	1	2	6	3	3	5
TCGA-G9-6332-01	2	1	1	1	6	3	8	7
TCGA-G9-6333-01	2	1	3	2	1	2	1	2
TCGA-G9-6336-01	2	1	1	1	5	3	4	7
TCGA-G9-6338-01	1	3	3	5	2	2	1	8
TCGA-G9-6339-01	1	3	3	5	2	1	6	6
TCGA-G9-6342-01	1	3	3	5	2	1	6	8
TCGA-G9-6343-01	2	2	1	1	5	3	4	9
TCGA-G9-6347-01	2	2	1	4	5	5	4	9
TCGA-G9-6348-01	2	2	1	1	5	5	5	9
TCGA-G9-6351-01	1	3	3	2	1	2	1	2
TCGA-G9-6353-01	1	3	3	5	2	1	6	6
TCGA-G9-6354-01	2	2	4	1	5	5	2	9
TCGA-G9-6356-01	1	3	3	5	2	1	6	6
TCGA-G9-6361-01	2	1	1	2	1	2	3	2

TCGA-G9-6362-01	2	2	4	3	6	3	4	7
TCGA-G9-6363-01	1	3	3	2	1	2	1	2
TCGA-G9-6364-01	2	2	4	1	5	5	2	9
TCGA-G9-6365-01	2	1	3	2	1	2	1	2
TCGA-G9-6366-01	2	2	1	1	6	3	4	7
TCGA-G9-6367-01	2	1	1	1	6	3	8	7
TCGA-G9-6369-01	2	1	1	2	6	6	3	5
TCGA-G9-6370-01	2	1	1	2	6	6	3	5
TCGA-G9-6371-01	1	3	3	5	2	1	6	6
TCGA-G9-6373-01	2	2	1	4	5	5	4	9
TCGA-G9-6377-01	2	1	1	2	6	6	8	5
TCGA-G9-6378-01	1	3	3	5	2	1	6	8
TCGA-G9-6379-01	2	2	1	4	5	5	4	9
TCGA-G9-6384-01	1	3	3	5	2	1	6	6
TCGA-G9-6385-01	2	1	1	2	1	2	1	2
TCGA-G9-6494-01	1	3	3	5	2	1	6	6
TCGA-G9-6496-01	1	3	3	5	2	1	6	6
TCGA-G9-6498-01	2	1	1	3	1	6	3	2
TCGA-G9-6499-01	2	1	2	1	6	3	8	7
TCGA-G9-7509-01	2	1	1	2	1	2	1	2
TCGA-G9-7510-01	2	1	2	4	3	7	5	4
TCGA-G9-7519-01	2	2	1	1	5	5	4	9
TCGA-G9-7521-01	2	1	2	4	3	7	5	4
TCGA-G9-7522-01	2	2	4	3	4	4	7	1
TCGA-G9-7523-01	1	3	3	5	2	1	6	6
TCGA-G9-7525-01	2	1	1	1	6	3	4	7
TCGA-G9-A9S0-01	2	1	2	4	3	7	5	4
TCGA-G9-A9S4-01	2	1	2	4	3	7	5	4
TCGA-G9-A9S7-01	2	2	4	1	5	5	2	9
TCGA-H9-7775-01	1	3	3	5	2	1	6	6
TCGA-H9-A6BX-01	1	3	3	5	2	1	6	6
TCGA-H9-A6BY-01	2	2	1	3	6	3	3	3
TCGA-HC-7075-01	2	2	1	1	6	3	8	3
TCGA-HC-7077-01	2	2	4	1	5	5	2	9
TCGA-HC-7078-01	2	1	1	1	6	3	8	7
TCGA-HC-7079-01	1	3	3	5	2	1	6	8
TCGA-HC-7080-01	2	2	4	3	5	5	4	3
TCGA-HC-7081-01	1	1	1	2	1	6	3	5
TCGA-HC-7209-01	1	3	3	5	2	1	6	8
TCGA-HC-7210-01	1	3	3	5	2	1	6	6
TCGA-HC-7211-01	1	3	3	5	2	1	6	6

TCGA-HC-7212-01	2	1	1	1	5	3	4	7
TCGA-HC-7213-01	2	1	2	4	3	7	4	4
TCGA-HC-7230-01	2	1	2	4	3	7	5	4
TCGA-HC-7231-01	2	1	1	1	6	3	8	7
TCGA-HC-7232-01	2	1	1	2	1	2	1	2
TCGA-HC-7233-01	2	1	1	2	6	6	3	5
TCGA-HC-7736-01	2	2	1	4	5	5	4	9
TCGA-HC-7737-01	2	1	1	2	6	6	3	5
TCGA-HC-7738-01	2	1	1	1	6	3	8	7
TCGA-HC-7740-01	2	1	1	2	1	2	1	2
TCGA-HC-7742-01	2	1	2	4	3	7	5	4
TCGA-HC-7744-01	2	2	1	1	5	5	4	3
TCGA-HC-7745-01	2	1	1	2	6	6	3	5
TCGA-HC-7747-01	1	3	3	5	2	1	6	6
TCGA-HC-7748-01	1	3	3	5	2	1	6	6
TCGA-HC-7749-01	2	2	1	3	6	3	8	3
TCGA-HC-7750-01	2	2	4	1	5	5	2	9
TCGA-HC-7752-01	1	3	3	5	2	1	6	6
TCGA-HC-7817-01	2	2	1	2	6	6	3	5
TCGA-HC-7818-01	1	3	3	5	2	1	6	6
TCGA-HC-7819-01	2	2	1	1	5	3	4	9
TCGA-HC-7820-01	2	2	1	3	6	3	8	3
TCGA-HC-7821-01	2	2	1	3	6	3	8	3
TCGA-HC-8213-01	1	3	3	5	2	1	6	6
TCGA-HC-8216-01	2	1	2	4	3	7	5	4
TCGA-HC-8256-01	2	1	2	4	3	3	4	7
TCGA-HC-8257-01	2	2	4	3	4	4	7	1
TCGA-HC-8258-01	2	2	1	1	5	3	4	9
TCGA-HC-8259-01	1	3	3	5	1	2	1	8
TCGA-HC-8260-01	2	1	1	1	6	3	4	7
TCGA-HC-8261-01	2	1	1	2	6	6	3	5
TCGA-HC-8262-01	2	1	1	1	6	3	8	7
TCGA-HC-8264-01	2	1	2	4	3	7	5	4
TCGA-HC-8265-01	2	1	1	1	6	3	8	7
TCGA-HC-8266-01	2	2	4	1	5	5	2	9
TCGA-HC-A48F-01	2	1	2	4	3	7	5	4
TCGA-HC-A4ZV-01	2	2	1	1	6	3	8	7
TCGA-HC-A631-01	1	1	2	4	3	7	5	4
TCGA-HC-A632-01	2	1	2	4	3	7	5	4
TCGA-HC-A6AL-01	2	2	4	1	5	5	2	9
TCGA-HC-A6AN-01	2	2	1	1	5	5	4	9

TCGA-HC-A6AO-01	2	2	4	3	6	6	3	5
TCGA-HC-A6AP-01	2	1	1	1	5	5	4	9
TCGA-HC-A6AQ-01	1	3	3	5	2	1	6	6
TCGA-HC-A6AS-01	1	3	3	5	2	1	6	8
TCGA-HC-A6HX-01	1	3	3	5	1	2	1	8
TCGA-HC-A6HY-01	1	3	3	5	1	2	1	8
TCGA-HC-A76W-01	1	1	3	2	1	2	1	2
TCGA-HC-A76X-01	2	2	1	4	5	5	4	9
TCGA-HC-A8CY-01	2	2	1	1	5	3	8	9
TCGA-HC-A8D0-01	2	1	1	1	5	3	8	7
TCGA-HC-A8D1-01	1	3	3	5	2	1	6	6
TCGA-HC-A9TE-01	1	1	2	4	3	7	5	4
TCGA-HC-A9TH-01	2	1	1	1	6	3	4	7
TCGA-HI-7168-01	2	2	1	2	6	6	3	5
TCGA-HI-7169-01	2	2	4	1	5	5	2	9
TCGA-HI-7170-01	2	2	4	1	5	5	2	9
TCGA-HI-7171-01	2	1	2	4	3	7	5	4
TCGA-J4-8198-01	1	3	3	5	1	2	1	8
TCGA-J4-8200-01	2	1	1	2	6	6	3	5
TCGA-J4-A67K-01	1	3	3	5	2	1	6	6
TCGA-J4-A67L-01	2	1	2	4	3	7	5	4
TCGA-J4-A67M-01	2	1	1	2	6	6	3	5
TCGA-J4-A67N-01	1	3	3	5	2	1	6	6
TCGA-J4-A67O-01	1	3	3	5	1	2	1	8
TCGA-J4-A67Q-01	2	2	1	1	5	5	4	3
TCGA-J4-A67R-01	2	1	1	2	6	6	3	5
TCGA-J4-A67S-01	1	3	3	5	2	1	6	8
TCGA-J4-A67T-01	1	3	3	5	2	1	6	8
TCGA-J4-A6G1-01	1	3	3	5	2	1	6	6
TCGA-J4-A6G3-01	2	1	1	1	6	3	8	7
TCGA-J4-A6M7-01	2	2	4	3	4	4	7	1
TCGA-J4-A83I-01	2	1	1	2	6	3	3	5
TCGA-J4-A83J-01	2	2	4	4	5	7	2	9
TCGA-J4-A83K-01	2	2	1	1	5	5	4	9
TCGA-J4-A83L-01	2	2	1	1	6	3	8	7
TCGA-J4-A83M-01	1	3	3	2	1	2	1	2
TCGA-J4-A83N-01	1	3	3	5	2	1	6	6
TCGA-J4-AATV-01	1	3	3	5	2	1	6	6
TCGA-J4-AATZ-01	2	2	1	1	5	3	4	7
TCGA-J4-AAU2-01	2	1	1	2	6	3	3	7
TCGA-J9-A52B-01	2	1	2	4	3	7	5	4

TCGA-J9-A52C-01	1	3	3	5	2	2	1	8
TCGA-J9-A52D-01	1	3	3	5	2	1	6	6
TCGA-J9-A52E-01	1	3	3	5	2	1	6	8
TCGA-J9-A8CK-01	1	1	2	4	3	7	5	4
TCGA-J9-A8CL-01	2	1	1	1	6	3	8	7
TCGA-J9-A8CM-01	2	2	4	3	5	5	2	3
TCGA-J9-A8CN-01	2	1	1	2	6	6	3	5
TCGA-J9-A8CP-01	1	3	3	5	2	1	6	6
TCGA-KC-A4BL-01	2	1	2	4	3	7	5	4
TCGA-KC-A4BN-01	1	3	3	5	2	1	6	6
TCGA-KC-A4BO-01	2	2	4	1	5	3	4	7
TCGA-KC-A4BR-01	2	1	1	1	6	3	8	7
TCGA-KC-A4BV-01	2	2	4	1	5	5	2	9
TCGA-KC-A7F3-01	1	3	3	5	2	1	6	8
TCGA-KC-A7F5-01	2	2	1	1	6	3	8	7
TCGA-KC-A7F6-01	2	1	2	4	3	3	4	4
TCGA-KC-A7FA-01	2	1	2	4	3	7	5	4
TCGA-KC-A7FD-01	2	1	1	1	6	3	8	7
TCGA-KC-A7FE-01	1	3	3	5	2	1	6	6
TCGA-KK-A59V-01	1	3	3	5	2	1	6	8
TCGA-KK-A59X-01	2	1	1	2	1	6	3	5
TCGA-KK-A59Y-01	2	2	1	2	6	3	8	3
TCGA-KK-A59Z-01	1	1	3	2	1	2	1	2
TCGA-KK-A5A1-01	2	1	1	1	6	3	8	7
TCGA-KK-A6DY-01	2	1	2	4	3	7	4	4
TCGA-KK-A6E0-01	1	3	3	5	2	1	6	6
TCGA-KK-A6E1-01	2	2	4	1	5	5	2	9
TCGA-KK-A6E2-01	2	1	1	1	6	3	8	7
TCGA-KK-A6E3-01	2	2	4	1	5	5	4	7
TCGA-KK-A6E4-01	2	2	1	1	5	3	4	9
TCGA-KK-A6E5-01	1	3	3	5	2	1	6	8
TCGA-KK-A6E6-01	2	2	4	3	4	4	7	1
TCGA-KK-A6E7-01	2	2	4	1	5	5	2	9
TCGA-KK-A6E8-01	1	1	3	2	1	2	1	2
TCGA-KK-A7AP-01	2	1	1	2	6	6	3	5
TCGA-KK-A7AQ-01	1	3	3	5	2	1	6	8
TCGA-KK-A7AU-01	1	1	2	4	3	7	5	4
TCGA-KK-A7AV-01	1	3	3	5	2	1	6	6
TCGA-KK-A7AW-01	1	3	3	5	2	1	6	6
TCGA-KK-A7AY-01	2	2	4	3	4	4	7	1
TCGA-KK-A7AZ-01	2	1	1	1	6	3	4	7

TCGA-KK-A7B0-01	2	1	1	1	5	5	4	7
TCGA-KK-A7B1-01	1	3	3	5	1	2	1	8
TCGA-KK-A7B2-01	2	1	1	1	6	3	8	7
TCGA-KK-A7B3-01	2	1	1	1	5	5	4	9
TCGA-KK-A7B4-01	2	1	2	4	3	7	5	4
TCGA-KK-A8I4-01	2	2	1	1	5	3	8	9
TCGA-KK-A8I5-01	1	3	3	5	2	1	6	6
TCGA-KK-A8I6-01	2	2	1	1	5	5	4	9
TCGA-KK-A8I7-01	2	2	4	1	5	5	2	9
TCGA-KK-A8I8-01	1	3	3	5	2	1	6	8
TCGA-KK-A8I9-01	2	2	1	4	5	5	4	9
TCGA-KK-A8IA-01	2	2	4	1	5	5	2	9
TCGA-KK-A8IB-01	1	3	3	5	2	1	6	8
TCGA-KK-A8IC-01	2	1	1	2	6	3	3	7
TCGA-KK-A8ID-01	2	2	4	1	5	5	2	9
TCGA-KK-A8IF-01	2	2	1	1	5	5	4	3
TCGA-KK-A8IG-01	2	1	2	4	3	7	5	4
TCGA-KK-A8IH-01	2	1	1	2	1	6	1	2
TCGA-KK-A8II-01	2	1	2	4	3	3	4	4
TCGA-KK-A8IJ-01	2	2	4	1	5	5	2	9
TCGA-KK-A8IK-01	2	1	1	2	6	6	3	5
TCGA-KK-A8IL-01	1	3	3	5	2	1	6	6
TCGA-KK-A8IM-01	1	3	3	5	2	1	6	6
TCGA-M7-A71Y-01	2	1	1	2	1	6	3	5
TCGA-M7-A71Z-01	1	3	3	5	2	1	6	6
TCGA-M7-A720-01	2	2	4	1	5	5	2	9
TCGA-M7-A721-01	1	3	3	5	2	1	6	8
TCGA-M7-A722-01	2	1	1	1	6	3	8	7
TCGA-M7-A723-01	2	2	1	1	5	3	4	7
TCGA-M7-A724-01	2	1	1	1	6	3	8	7
TCGA-M7-A725-01	2	2	1	1	5	5	4	9
TCGA-MG-AAMC-01	1	3	3	5	2	1	1	8
TCGA-QU-A6IL-01	2	2	4	3	5	5	4	3
TCGA-QU-A6IM-01	2	1	1	1	6	3	8	7
TCGA-QU-A6IN-01	2	1	1	1	6	3	8	7
TCGA-QU-A6IO-01	2	2	1	3	6	5	8	3
TCGA-QU-A6IP-01	2	1	1	2	1	2	1	2
TCGA-SU-A7E7-01	1	3	3	5	2	2	1	8
TCGA-TK-A8OK-01	1	3	3	5	1	2	1	2
TCGA-TP-A8TT-01	2	1	1	2	6	6	3	5
TCGA-TP-A8TV-01	2	1	1	1	6	3	3	7

TCGA-V1-A8MF-01	2	2	1	1	5	3	8	9
TCGA-V1-A8MG-01	1	3	3	5	2	1	6	6
TCGA-V1-A8MJ-01	2	1	2	4	3	7	5	4
TCGA-V1-A8MK-01	1	3	3	5	2	1	6	6
TCGA-V1-A8ML-01	1	3	3	5	2	1	6	6
TCGA-V1-A8MM-01	1	3	3	5	2	1	6	6
TCGA-V1-A8MU-01	2	1	1	1	6	3	8	7
TCGA-V1-A8WL-01	2	2	1	1	5	5	4	3
TCGA-V1-A8WN-01	2	1	4	1	5	3	4	7
TCGA-V1-A8WS-01	2	2	1	1	5	3	4	7
TCGA-V1-A8WV-01	2	1	1	1	6	3	8	7
TCGA-V1-A8WW-01	2	1	2	4	6	3	8	7
TCGA-V1-A8X3-01	1	3	3	5	2	1	1	8
TCGA-V1-A9O5-01	1	1	2	4	3	7	5	4
TCGA-V1-A9O7-01	2	2	1	1	6	3	8	3
TCGA-V1-A9O9-01	2	1	1	1	6	3	8	7
TCGA-V1-A9OA-01	1	3	3	5	2	1	6	6
TCGA-V1-A9OF-01	1	3	3	5	2	1	6	8
TCGA-V1-A9OH-01	2	2	1	1	6	3	8	3
TCGA-V1-A9OL-01	1	3	3	5	2	1	6	6
TCGA-V1-A9OQ-01	2	1	2	1	6	3	8	7
TCGA-V1-A9OT-01	1	3	3	5	2	1	6	6
TCGA-V1-A9OX-01	2	1	1	2	6	6	3	5
TCGA-V1-A9OY-01	1	3	3	5	2	1	1	8
TCGA-V1-A9Z7-01	2	2	1	1	5	5	4	9
TCGA-V1-A9Z8-01	1	3	3	5	2	1	6	8
TCGA-V1-A9Z9-01	2	2	4	1	5	5	2	9
TCGA-V1-A9ZG-01	2	1	1	2	6	6	3	7
TCGA-V1-A9ZI-01	2	1	2	4	3	7	5	4
TCGA-V1-A9ZK-01	2	2	4	3	5	5	8	3
TCGA-V1-A9ZR-01	1	3	3	5	2	1	6	6
TCGA-VN-A88I-01	1	3	3	5	1	2	1	8
TCGA-VN-A88K-01	2	2	1	1	5	3	4	3
TCGA-VN-A88L-01	2	1	3	2	1	2	1	2
TCGA-VN-A88M-01	1	3	3	5	1	2	1	8
TCGA-VN-A88N-01	2	2	1	2	6	6	3	5
TCGA-VN-A88O-01	1	3	3	5	2	1	6	8
TCGA-VN-A88P-01	1	3	3	5	2	1	6	6
TCGA-VN-A88Q-01	2	1	1	2	6	6	3	5
TCGA-VN-A88R-01	2	1	1	2	6	6	3	5
TCGA-VN-A943-01	1	3	3	5	2	1	6	6

TCGA-VP-A872-01	2	1	2	4	3	7	5	4
TCGA-VP-A875-01	2	1	1	2	6	6	3	5
TCGA-VP-A876-01	2	1	2	4	3	7	4	4
TCGA-VP-A878-01	2	2	1	1	6	3	8	3
TCGA-VP-A879-01	2	1	1	2	6	6	8	7
TCGA-VP-A87B-01	2	1	1	2	6	6	3	5
TCGA-VP-A87C-01	1	1	3	5	1	2	1	2
TCGA-VP-A87D-01	1	1	2	4	3	3	4	4
TCGA-VP-A87E-01	1	3	3	5	2	1	6	6
TCGA-VP-A87H-01	2	1	1	1	6	3	8	7
TCGA-VP-A87J-01	1	3	3	5	2	1	6	6
TCGA-VP-A87K-01	2	1	1	1	6	3	8	7
TCGA-VP-AA1N-01	2	1	1	1	6	3	8	7
TCGA-WW-A8ZI-01	2	2	4	1	5	5	2	9
TCGA-X4-A8KQ-01	2	2	4	1	5	5	2	9
TCGA-X4-A8KS-01	2	1	3	2	1	2	1	2
TCGA-XA-A8JR-01	1	3	3	5	2	1	6	6
TCGA-XJ-A83F-01	2	1	3	5	1	2	1	8
TCGA-XJ-A83G-01	2	2	4	3	4	4	7	1
TCGA-XJ-A83H-01	1	1	3	2	1	2	1	2
TCGA-XJ-A9DI-01	2	1	2	4	3	7	5	4
TCGA-XJ-A9DK-01	2	2	4	1	5	7	2	9
TCGA-XJ-A9DQ-01	1	3	3	5	2	2	1	8
TCGA-XJ-A9DX-01	2	1	1	2	6	6	3	7
TCGA-XK-AAIR-01	2	1	3	2	1	2	1	2
TCGA-XK-AAIV-01	2	1	1	2	1	2	1	2
TCGA-XK-AAIW-01	1	3	3	5	2	1	6	8
TCGA-XK-AAJ3-01	2	1	1	1	6	3	8	7
TCGA-XK-AAJA-01	1	3	3	5	2	1	6	8
TCGA-XK-AAJP-01	2	2	1	1	6	3	8	3
TCGA-XK-AAJR-01	1	1	3	2	1	2	1	2
TCGA-XK-AAJT-01	1	3	3	5	2	1	6	6
TCGA-XK-AAJU-01	1	3	3	5	1	2	1	8
TCGA-XK-AAK1-01	2	1	1	1	6	3	8	7
TCGA-XQ-A8TA-01	2	2	4	1	5	5	2	9
TCGA-XQ-A8TB-01	2	2	4	1	5	5	2	9
TCGA-Y6-A8TL-01	2	2	1	1	6	3	8	7
TCGA-Y6-A9XI-01	2	2	4	3	4	4	7	1
TCGA-YJ-A8SW-01	1	3	3	5	2	1	6	6
TCGA-YL-A8HJ-01	2	2	4	1	5	5	2	9
TCGA-YL-A8HK-01	2	1	2	4	3	7	5	4

TCGA-YL-A8HL-01	2	1	2	4	3	7	5	4
TCGA-YL-A8HM-01	2	1	2	4	3	7	5	4
TCGA-YL-A8HO-01	2	1	1	2	1	6	3	5
TCGA-YL-A8S8-01	2	2	1	1	5	5	4	9
TCGA-YL-A8S9-01	2	2	4	1	5	5	2	9
TCGA-YL-A8SA-01	1	3	3	5	2	1	6	6
TCGA-YL-A8SB-01	2	1	2	4	3	7	4	4
TCGA-YL-A8SC-01	2	2	4	1	5	5	4	3
TCGA-YL-A8SF-01	2	2	4	1	5	5	2	9
TCGA-YL-A8SH-01	2	2	1	1	5	3	8	9
TCGA-YL-A8SI-01	1	3	3	5	2	1	6	6
TCGA-YL-A8SJ-01	2	1	2	4	3	7	5	4
TCGA-YL-A8SK-01	1	3	3	5	2	1	6	8
TCGA-YL-A8SL-01	2	2	4	3	4	4	7	1
TCGA-YL-A8SO-01	2	2	1	1	5	5	4	9
TCGA-YL-A8SP-01	2	2	1	3	6	3	8	3
TCGA-YL-A8SQ-01	2	1	1	2	6	6	3	5
TCGA-YL-A8SR-01	2	1	1	2	6	3	3	5
TCGA-YL-A9WH-01	2	1	2	4	3	7	5	4
TCGA-YL-A9WI-01	2	2	1	1	5	5	4	9
TCGA-YL-A9WJ-01	1	3	3	5	2	1	6	6
TCGA-YL-A9WK-01	2	1	2	4	3	7	5	4
TCGA-YL-A9WL-01	1	3	3	5	2	1	6	6
TCGA-YL-A9WX-01	1	1	2	4	3	7	5	4
TCGA-YL-A9WY-01	2	1	2	4	3	7	5	4
TCGA-ZG-A8QW-01	2	2	4	1	5	5	2	9
TCGA-ZG-A8QX-01	1	3	3	5	2	1	6	6
TCGA-ZG-A8QY-01	2	2	1	4	5	5	4	9
TCGA-ZG-A8QZ-01	1	3	3	5	1	2	1	8
TCGA-ZG-A9KY-01	2	1	1	2	6	6	3	7
TCGA-ZG-A9L0-01	1	1	2	4	3	7	5	4
TCGA-ZG-A9L1-01	1	1	2	4	3	7	5	4
TCGA-ZG-A9L2-01	2	2	4	1	5	5	2	9
TCGA-ZG-A9L4-01	2	1	1	2	1	2	1	2
TCGA-ZG-A9L5-01	2	2	4	1	5	5	2	9
TCGA-ZG-A9L6-01	2	2	1	1	6	3	8	7
TCGA-ZG-A9L9-01	1	3	3	5	2	1	6	6
TCGA-ZG-A9LB-01	2	1	2	4	6	3	8	7
TCGA-ZG-A9LM-01	2	1	2	4	3	7	5	4
TCGA-ZG-A9LN-01	2	2	4	3	4	4	7	1
TCGA-ZG-A9LS-01	2	2	4	1	5	5	2	9

TCGA-ZG-A9LU-01	1	3	3	5	2	1	6	6
TCGA-ZG-A9LY-01	2	2	4	1	5	5	2	9
TCGA-ZG-A9LZ-01	1	1	1	1	6	6	3	5
TCGA-ZG-A9M4-01	2	1	2	4	3	7	5	4
TCGA-ZG-A9MC-01	1	3	3	5	2	1	6	6
TCGA-ZG-A9N3-01	2	1	1	2	6	6	8	5
TCGA-ZG-A9ND-01	2	1	3	2	1	2	1	2
TCGA-ZG-A9NI-01	2	1	1	2	6	6	8	7

Table S2 List of samples with 3 subtypes and silhouette width

Sample name	Subtype	Silhouette width
TCGA-2A-A8VT-01	1	0.526173114
TCGA-2A-A8VV-01	1	0.761408761
TCGA-2A-A8VX-01	1	0.784589591
TCGA-2A-A8W1-01	1	0.814626494
TCGA-CH-5741-01	1	0.833070894
TCGA-CH-5743-01	1	0.630107793
TCGA-CH-5745-01	1	0.779773099
TCGA-CH-5750-01	1	0.465009732
TCGA-CH-5751-01	1	0.787079248
TCGA-CH-5752-01	1	0.803495028
TCGA-CH-5754-01	1	0.789855024
TCGA-CH-5761-01	1	0.735255878
TCGA-CH-5762-01	1	0.580771083
TCGA-CH-5764-01	1	0.757924634
TCGA-CH-5768-01	1	0.852773904
TCGA-CH-5772-01	1	0.815097573
TCGA-CH-5788-01	1	0.764553946
TCGA-CH-5792-01	1	0.760115956
TCGA-EJ-5494-01	1	0.736803098
TCGA-EJ-5498-01	1	0.698749531
TCGA-EJ-5499-01	1	0.722743966
TCGA-EJ-5504-01	1	0.628599417
TCGA-EJ-5507-01	1	0.710269348
TCGA-EJ-5509-01	1	0.495883391
TCGA-EJ-5512-01	1	0.541798607
TCGA-EJ-5514-01	1	0.605008517
TCGA-EJ-5521-01	1	0.720271037
TCGA-EJ-5525-01	1	0.802612271
TCGA-EJ-5526-01	1	0.698735997
TCGA-EJ-5530-01	1	0.756849468
TCGA-EJ-5531-01	1	0.696355424
TCGA-EJ-7115-01	1	0.692849141
TCGA-EJ-7123-01	1	0.598689964
TCGA-EJ-7315-01	1	0.747521436
TCGA-EJ-7318-01	1	0.732466025
TCGA-EJ-7321-01	1	0.577058384
TCGA-EJ-7328-01	1	0.808606669
TCGA-EJ-7331-01	1	0.819773088
TCGA-EJ-7781-01	1	0.828412912

TCGA-EJ-7784-01	1	0.801372621
TCGA-EJ-7785-01	1	0.822014272
TCGA-EJ-7789-01	1	0.828031024
TCGA-EJ-7793-01	1	0.631523625
TCGA-EJ-7797-01	1	0.715373117
TCGA-EJ-8469-01	1	0.785998856
TCGA-EJ-8472-01	1	0.792585273
TCGA-EJ-8474-01	1	0.667994982
TCGA-EJ-A46E-01	1	0.291166713
TCGA-EJ-A46F-01	1	0.477463468
TCGA-EJ-A65D-01	1	0.699949245
TCGA-EJ-A65E-01	1	0.338565301
TCGA-EJ-A7NH-01	1	0.715415914
TCGA-EJ-A7NJ-01	1	0.573419307
TCGA-EJ-A7NN-01	1	0.42641299
TCGA-EJ-AB27-01	1	0.317525273
TCGA-FC-A5OB-01	1	0.81187667
TCGA-FC-A66V-01	1	0.700263715
TCGA-FC-A6HD-01	1	0.847585006
TCGA-FC-A8O0-01	1	0.737018037
TCGA-G9-6329-01	1	0.538908582
TCGA-G9-6332-01	1	0.664704593
TCGA-G9-6333-01	1	0.771550988
TCGA-G9-6336-01	1	0.664234814
TCGA-G9-6361-01	1	0.599792317
TCGA-G9-6365-01	1	0.595757564
TCGA-G9-6367-01	1	0.688830731
TCGA-G9-6369-01	1	0.807992971
TCGA-G9-6370-01	1	0.729555102
TCGA-G9-6377-01	1	0.824164157
TCGA-G9-6385-01	1	0.762483866
TCGA-G9-6498-01	1	0.394514762
TCGA-G9-6499-01	1	0.786812648
TCGA-G9-7509-01	1	0.80170814
TCGA-G9-7510-01	1	0.690922217
TCGA-G9-7521-01	1	0.730222854
TCGA-G9-7525-01	1	0.718176446
TCGA-G9-A9S0-01	1	0.68437256
TCGA-G9-A9S4-01	1	0.602959773
TCGA-HC-7078-01	1	0.750331511
TCGA-HC-7081-01	1	0.134323058

TCGA-HC-7212-01	1	0.629298391
TCGA-HC-7213-01	1	0.787634601
TCGA-HC-7230-01	1	0.709962077
TCGA-HC-7231-01	1	0.794906463
TCGA-HC-7232-01	1	0.700182399
TCGA-HC-7233-01	1	0.760911064
TCGA-HC-7737-01	1	0.718066991
TCGA-HC-7738-01	1	0.756931021
TCGA-HC-7740-01	1	0.795338437
TCGA-HC-7742-01	1	0.731926121
TCGA-HC-7745-01	1	0.741118028
TCGA-HC-8216-01	1	0.62768948
TCGA-HC-8256-01	1	0.812243851
TCGA-HC-8260-01	1	0.590517318
TCGA-HC-8261-01	1	0.749810526
TCGA-HC-8262-01	1	0.708440529
TCGA-HC-8264-01	1	0.675311365
TCGA-HC-8265-01	1	0.754079949
TCGA-HC-A48F-01	1	0.752321183
TCGA-HC-A631-01	1	0.638558665
TCGA-HC-A632-01	1	0.574259587
TCGA-HC-A6AP-01	1	0.670719067
TCGA-HC-A76W-01	1	0.240880827
TCGA-HC-A8D0-01	1	0.468478945
TCGA-HC-A9TE-01	1	0.545882911
TCGA-HC-A9TH-01	1	0.850469597
TCGA-HI-7171-01	1	0.761979597
TCGA-J4-8200-01	1	0.76722962
TCGA-J4-A67L-01	1	0.702594131
TCGA-J4-A67M-01	1	0.746901902
TCGA-J4-A67R-01	1	0.763615873
TCGA-J4-A6G3-01	1	0.856244947
TCGA-J4-A83I-01	1	0.709074545
TCGA-J4-AAU2-01	1	0.450902939
TCGA-J9-A52B-01	1	0.721992125
TCGA-J9-A8CK-01	1	0.668952063
TCGA-J9-A8CL-01	1	0.749398088
TCGA-J9-A8CN-01	1	0.823898724
TCGA-KC-A4BL-01	1	0.741282434
TCGA-KC-A4BR-01	1	0.531156799
TCGA-KC-A7F6-01	1	0.792504118

TCGA-KC-A7FA-01	1	0.594602158
TCGA-KC-A7FD-01	1	0.818851211
TCGA-KK-A59X-01	1	0.651854319
TCGA-KK-A59Z-01	1	0.310680089
TCGA-KK-A5A1-01	1	0.827499968
TCGA-KK-A6DY-01	1	0.810286708
TCGA-KK-A6E2-01	1	0.706391001
TCGA-KK-A6E8-01	1	0.216888442
TCGA-KK-A7AP-01	1	0.837811808
TCGA-KK-A7AU-01	1	0.640015944
TCGA-KK-A7AZ-01	1	0.785258822
TCGA-KK-A7B0-01	1	0.560387484
TCGA-KK-A7B2-01	1	0.788213175
TCGA-KK-A7B3-01	1	0.618514174
TCGA-KK-A7B4-01	1	0.704506374
TCGA-KK-A8IC-01	1	0.436900055
TCGA-KK-A8IG-01	1	0.707502944
TCGA-KK-A8IH-01	1	0.538825358
TCGA-KK-A8II-01	1	0.815179382
TCGA-KK-A8IK-01	1	0.786365349
TCGA-M7-A71Y-01	1	0.699263273
TCGA-M7-A722-01	1	0.765293491
TCGA-M7-A724-01	1	0.621242655
TCGA-QU-A6IM-01	1	0.70269211
TCGA-QU-A6IN-01	1	0.648654978
TCGA-QU-A6IP-01	1	0.649999707
TCGA-TP-A8TT-01	1	0.80184369
TCGA-TP-A8TV-01	1	0.62615959
TCGA-V1-A8MJ-01	1	0.698887439
TCGA-V1-A8MU-01	1	0.718989326
TCGA-V1-A8WN-01	1	0.460685019
TCGA-V1-A8WV-01	1	0.536585723
TCGA-V1-A8WW-01	1	0.788611964
TCGA-V1-A9O5-01	1	0.638394345
TCGA-V1-A9O9-01	1	0.812257577
TCGA-V1-A9OQ-01	1	0.834670384
TCGA-V1-A9OX-01	1	0.843324784
TCGA-V1-A9ZG-01	1	0.777860146
TCGA-V1-A9ZI-01	1	0.707678713
TCGA-VN-A88L-01	1	0.374446876
TCGA-VN-A88Q-01	1	0.718209398

TCGA-VN-A88R-01	1	0.767397755
TCGA-VP-A872-01	1	0.751138683
TCGA-VP-A875-01	1	0.846232802
TCGA-VP-A876-01	1	0.781827199
TCGA-VP-A879-01	1	0.764418738
TCGA-VP-A87B-01	1	0.624261948
TCGA-VP-A87C-01	1	0.00042165
TCGA-VP-A87D-01	1	0.509107691
TCGA-VP-A87H-01	1	0.728572103
TCGA-VP-A87K-01	1	0.735809744
TCGA-VP-AA1N-01	1	0.783705576
TCGA-X4-A8KS-01	1	0.565186207
TCGA-XJ-A83F-01	1	0.183821211
TCGA-XJ-A83H-01	1	0.353677631
TCGA-XJ-A9DI-01	1	0.735143567
TCGA-XJ-A9DX-01	1	0.6334439
TCGA-XK-AAIR-01	1	0.268133507
TCGA-XK-AAIV-01	1	0.629134143
TCGA-XK-AAJ3-01	1	0.636313773
TCGA-XK-AAJR-01	1	0.349206447
TCGA-XK-AAK1-01	1	0.650932808
TCGA-YL-A8HK-01	1	0.739518755
TCGA-YL-A8HL-01	1	0.747302729
TCGA-YL-A8HM-01	1	0.721227452
TCGA-YL-A8HO-01	1	0.821352606
TCGA-YL-A8SB-01	1	0.780528761
TCGA-YL-A8SJ-01	1	0.703500786
TCGA-YL-A8SQ-01	1	0.721105713
TCGA-YL-A8SR-01	1	0.710781051
TCGA-YL-A9WH-01	1	0.703171118
TCGA-YL-A9WK-01	1	0.65814183
TCGA-YL-A9WX-01	1	0.668175457
TCGA-YL-A9WY-01	1	0.735453249
TCGA-ZG-A9KY-01	1	0.792421313
TCGA-ZG-A9L0-01	1	0.555666902
TCGA-ZG-A9L1-01	1	0.614911111
TCGA-ZG-A9L4-01	1	0.827209501
TCGA-ZG-A9LB-01	1	0.723398109
TCGA-ZG-A9LM-01	1	0.687732469
TCGA-ZG-A9LZ-01	1	0.572916018
TCGA-ZG-A9M4-01	1	0.700961106

TCGA-ZG-A9N3-01	1	0.684849974
TCGA-ZG-A9ND-01	1	0.551549402
TCGA-ZG-A9NI-01	1	0.707424198
TCGA-2A-A8VL-01	2	-0.336559499
TCGA-2A-A8VO-01	2	0.205632777
TCGA-2A-AAYF-01	2	0.179772911
TCGA-4L-AA1F-01	2	-0.419367693
TCGA-CH-5737-01	2	-0.35577367
TCGA-CH-5738-01	2	0.492885668
TCGA-CH-5740-01	2	0.007222333
TCGA-CH-5748-01	2	0.114455665
TCGA-CH-5765-01	2	0.387396377
TCGA-CH-5769-01	2	0.187687893
TCGA-CH-5771-01	2	-0.43744629
TCGA-CH-5789-01	2	0.421172472
TCGA-CH-5791-01	2	0.028931678
TCGA-CH-5794-01	2	0.492400301
TCGA-EJ-5495-01	2	-0.251516724
TCGA-EJ-5496-01	2	0.027063697
TCGA-EJ-5501-01	2	-0.40902524
TCGA-EJ-5503-01	2	0.034437092
TCGA-EJ-5505-01	2	0.01391646
TCGA-EJ-5506-01	2	-0.41838678
TCGA-EJ-5508-01	2	0.297054453
TCGA-EJ-5511-01	2	-0.222157248
TCGA-EJ-5518-01	2	-0.005979449
TCGA-EJ-5519-01	2	0.529477836
TCGA-EJ-5522-01	2	-0.172822764
TCGA-EJ-5527-01	2	0.068757778
TCGA-EJ-7218-01	2	-0.418311022
TCGA-EJ-7325-01	2	0.141100404
TCGA-EJ-7783-01	2	-0.490500032
TCGA-EJ-7786-01	2	-0.076265087
TCGA-EJ-7791-01	2	-0.021665845
TCGA-EJ-A46G-01	2	0.233101435
TCGA-EJ-A46H-01	2	-0.440398339
TCGA-EJ-A65B-01	2	-0.05629669
TCGA-EJ-A65F-01	2	-0.315912491
TCGA-EJ-A6RC-01	2	0.525022666
TCGA-EJ-A7NF-01	2	0.17688543
TCGA-EJ-A7NM-01	2	0.024110938

TCGA-EJ-A8FN-01	2	0.310283708
TCGA-EJ-A8FS-01	2	-0.171966028
TCGA-EJ-A8FU-01	2	0.078127155
TCGA-FC-7961-01	2	0.183929327
TCGA-G9-6343-01	2	-0.377811044
TCGA-G9-6347-01	2	0.13289558
TCGA-G9-6348-01	2	-0.269080781
TCGA-G9-6354-01	2	0.25310173
TCGA-G9-6362-01	2	-0.069185247
TCGA-G9-6364-01	2	0.271388877
TCGA-G9-6366-01	2	-0.435303807
TCGA-G9-6373-01	2	0.031695662
TCGA-G9-6379-01	2	-0.215513007
TCGA-G9-7519-01	2	-0.414063819
TCGA-G9-7522-01	2	0.310017741
TCGA-G9-A9S7-01	2	0.261422937
TCGA-H9-A6BY-01	2	0.007940655
TCGA-HC-7075-01	2	-0.416424201
TCGA-HC-7077-01	2	-0.206765393
TCGA-HC-7080-01	2	0.374888132
TCGA-HC-7736-01	2	-0.02818355
TCGA-HC-7744-01	2	0.280160044
TCGA-HC-7749-01	2	0.017973311
TCGA-HC-7750-01	2	0.085485069
TCGA-HC-7817-01	2	-0.39813761
TCGA-HC-7819-01	2	-0.118220813
TCGA-HC-7820-01	2	0.131686238
TCGA-HC-7821-01	2	0.135646869
TCGA-HC-8257-01	2	0.294104991
TCGA-HC-8258-01	2	-0.162291345
TCGA-HC-8266-01	2	-0.211208039
TCGA-HC-A4ZV-01	2	-0.331844846
TCGA-HC-A6AL-01	2	0.247795138
TCGA-HC-A6AN-01	2	-0.44617077
TCGA-HC-A6AO-01	2	-0.284637402
TCGA-HC-A76X-01	2	-0.113376489
TCGA-HC-A8CY-01	2	-0.447902298
TCGA-HI-7168-01	2	-0.36479096
TCGA-HI-7169-01	2	0.236229556
TCGA-HI-7170-01	2	0.204768027
TCGA-J4-A67Q-01	2	0.458232626

TCGA-J4-A6M7-01	2	0.337281328
TCGA-J4-A83J-01	2	-0.272259916
TCGA-J4-A83K-01	2	-0.369717176
TCGA-J4-A83L-01	2	-0.189061776
TCGA-J4-AATZ-01	2	-0.33589304
TCGA-J9-A8CM-01	2	0.433528791
TCGA-KC-A4BO-01	2	-0.343273824
TCGA-KC-A4BV-01	2	-0.124655252
TCGA-KC-A7F5-01	2	-0.443325042
TCGA-KK-A59Y-01	2	-0.209681812
TCGA-KK-A6E1-01	2	0.318284243
TCGA-KK-A6E3-01	2	-0.342798958
TCGA-KK-A6E4-01	2	-0.375396074
TCGA-KK-A6E6-01	2	0.363082927
TCGA-KK-A6E7-01	2	0.246018041
TCGA-KK-A7AY-01	2	0.361987014
TCGA-KK-A8I4-01	2	-0.267066079
TCGA-KK-A8I6-01	2	-0.063752676
TCGA-KK-A8I7-01	2	0.255780699
TCGA-KK-A8I9-01	2	0.046024942
TCGA-KK-A8IA-01	2	0.328193744
TCGA-KK-A8ID-01	2	0.205103272
TCGA-KK-A8IF-01	2	0.313025546
TCGA-KK-A8IJ-01	2	0.246077419
TCGA-M7-A720-01	2	0.215083955
TCGA-M7-A723-01	2	-0.31548762
TCGA-M7-A725-01	2	-0.153044706
TCGA-QU-A6IL-01	2	0.514483407
TCGA-QU-A6IO-01	2	0.382480979
TCGA-V1-A8MF-01	2	-0.315266082
TCGA-V1-A8WL-01	2	0.26505927
TCGA-V1-A8WS-01	2	-0.267016459
TCGA-V1-A9O7-01	2	0.094686388
TCGA-V1-A9OH-01	2	-0.326562657
TCGA-V1-A9Z7-01	2	-0.182579967
TCGA-V1-A9Z9-01	2	0.266341351
TCGA-V1-A9ZK-01	2	0.394795451
TCGA-VN-A88K-01	2	0.240604497
TCGA-VN-A88N-01	2	-0.18480673
TCGA-VP-A878-01	2	0.209600338
TCGA-WW-A8ZI-01	2	0.063249302

TCGA-X4-A8KQ-01	2	0.224824156
TCGA-XJ-A83G-01	2	0.306871103
TCGA-XJ-A9DK-01	2	0.323535969
TCGA-XK-AAJP-01	2	-0.142252933
TCGA-XQ-A8TA-01	2	0.013974138
TCGA-XQ-A8TB-01	2	0.269311276
TCGA-Y6-A8TL-01	2	-0.358738929
TCGA-Y6-A9XI-01	2	0.406143405
TCGA-YL-A8HJ-01	2	0.167374778
TCGA-YL-A8S8-01	2	0.07190116
TCGA-YL-A8S9-01	2	0.380399146
TCGA-YL-A8SC-01	2	0.505404491
TCGA-YL-A8SF-01	2	0.242686406
TCGA-YL-A8SH-01	2	-0.490623898
TCGA-YL-A8SL-01	2	0.335283244
TCGA-YL-A8SO-01	2	-0.096451116
TCGA-YL-A8SP-01	2	0.027815142
TCGA-YL-A9WI-01	2	-0.185951839
TCGA-ZG-A8QW-01	2	0.241042013
TCGA-ZG-A8QY-01	2	-0.214810568
TCGA-ZG-A9L2-01	2	0.192694144
TCGA-ZG-A9L5-01	2	0.272043782
TCGA-ZG-A9L6-01	2	-0.272646444
TCGA-ZG-A9LN-01	2	0.318596801
TCGA-ZG-A9LS-01	2	0.261309236
TCGA-ZG-A9LY-01	2	0.291922187
TCGA-2A-A8W3-01	3	0.920406902
TCGA-2A-AAYO-01	3	0.852860937
TCGA-2A-AAYU-01	3	0.742661387
TCGA-CH-5739-01	3	0.455497123
TCGA-CH-5744-01	3	0.927623982
TCGA-CH-5746-01	3	0.829964069
TCGA-CH-5753-01	3	0.785950532
TCGA-CH-5763-01	3	0.686118414
TCGA-CH-5766-01	3	0.320111528
TCGA-CH-5767-01	3	0.766558095
TCGA-CH-5790-01	3	0.891161949
TCGA-EJ-5497-01	3	0.346541848
TCGA-EJ-5502-01	3	0.858401177
TCGA-EJ-5510-01	3	0.737867857
TCGA-EJ-5515-01	3	0.908419048

TCGA-EJ-5516-01	3	0.851466887
TCGA-EJ-5517-01	3	0.921928367
TCGA-EJ-5524-01	3	0.926032544
TCGA-EJ-5532-01	3	0.921556464
TCGA-EJ-5542-01	3	0.886561639
TCGA-EJ-7125-01	3	0.929055085
TCGA-EJ-7312-01	3	0.909800917
TCGA-EJ-7314-01	3	0.930090786
TCGA-EJ-7317-01	3	0.871498547
TCGA-EJ-7327-01	3	0.917883472
TCGA-EJ-7330-01	3	0.745237344
TCGA-EJ-7782-01	3	0.905359991
TCGA-EJ-7788-01	3	0.928949084
TCGA-EJ-7792-01	3	0.82404122
TCGA-EJ-7794-01	3	0.900372364
TCGA-EJ-8468-01	3	0.387787637
TCGA-EJ-8470-01	3	0.914813049
TCGA-EJ-A46B-01	3	0.17658493
TCGA-EJ-A46D-01	3	0.918852127
TCGA-EJ-A46I-01	3	0.92986405
TCGA-EJ-A65G-01	3	0.821185212
TCGA-EJ-A65J-01	3	0.897598163
TCGA-EJ-A65M-01	3	0.906049366
TCGA-EJ-A6RA-01	3	0.931054079
TCGA-EJ-A7NG-01	3	0.906371549
TCGA-EJ-A7NK-01	3	0.853837167
TCGA-EJ-A8FO-01	3	0.899829863
TCGA-EJ-A8FP-01	3	0.913138575
TCGA-EJ-AB20-01	3	0.929203316
TCGA-FC-7708-01	3	0.35584207
TCGA-FC-A4JI-01	3	0.843087347
TCGA-G9-6338-01	3	0.88444932
TCGA-G9-6339-01	3	0.930022648
TCGA-G9-6342-01	3	0.756261185
TCGA-G9-6351-01	3	0.293877709
TCGA-G9-6353-01	3	0.904623648
TCGA-G9-6356-01	3	0.884596146
TCGA-G9-6363-01	3	0.242553103
TCGA-G9-6371-01	3	0.927291529
TCGA-G9-6378-01	3	0.636551595
TCGA-G9-6384-01	3	0.92937518

TCGA-G9-6494-01	3	0.91345904
TCGA-G9-6496-01	3	0.927533746
TCGA-G9-7523-01	3	0.922676376
TCGA-H9-7775-01	3	0.928233548
TCGA-H9-A6BX-01	3	0.893207272
TCGA-HC-7079-01	3	0.912615197
TCGA-HC-7209-01	3	0.893429329
TCGA-HC-7210-01	3	0.929752112
TCGA-HC-7211-01	3	0.882805619
TCGA-HC-7747-01	3	0.914718964
TCGA-HC-7748-01	3	0.908042303
TCGA-HC-7752-01	3	0.928204456
TCGA-HC-7818-01	3	0.918465328
TCGA-HC-8213-01	3	0.912684408
TCGA-HC-8259-01	3	0.677942371
TCGA-HC-A6AQ-01	3	0.895976722
TCGA-HC-A6AS-01	3	0.805419347
TCGA-HC-A6HX-01	3	0.461394649
TCGA-HC-A6HY-01	3	0.140255687
TCGA-HC-A8D1-01	3	0.930957927
TCGA-J4-8198-01	3	0.405720131
TCGA-J4-A67K-01	3	0.917741736
TCGA-J4-A67N-01	3	0.897391236
TCGA-J4-A67O-01	3	0.441635067
TCGA-J4-A67S-01	3	0.749530402
TCGA-J4-A67T-01	3	0.928097775
TCGA-J4-A6G1-01	3	0.885692737
TCGA-J4-A83M-01	3	0.587151061
TCGA-J4-A83N-01	3	0.900990327
TCGA-J4-AATV-01	3	0.877420487
TCGA-J9-A52C-01	3	0.886974562
TCGA-J9-A52D-01	3	0.8996399
TCGA-J9-A52E-01	3	0.923522643
TCGA-J9-A8CP-01	3	0.932045065
TCGA-KC-A4BN-01	3	0.837235244
TCGA-KC-A7F3-01	3	0.677066672
TCGA-KC-A7FE-01	3	0.880948526
TCGA-KK-A59V-01	3	0.918377002
TCGA-KK-A6E0-01	3	0.892603139
TCGA-KK-A6E5-01	3	0.927849429
TCGA-KK-A7AQ-01	3	0.80475159

TCGA-KK-A7AV-01	3	0.85121445
TCGA-KK-A7AW-01	3	0.925093537
TCGA-KK-A7B1-01	3	0.376742711
TCGA-KK-A8I5-01	3	0.886792777
TCGA-KK-A8I8-01	3	0.829178074
TCGA-KK-A8IB-01	3	0.74026911
TCGA-KK-A8IL-01	3	0.918655494
TCGA-KK-A8IM-01	3	0.931393051
TCGA-M7-A71Z-01	3	0.90194116
TCGA-M7-A721-01	3	0.86231902
TCGA-MG-AAMC-01	3	0.798667247
TCGA-SU-A7E7-01	3	0.58096008
TCGA-TK-A8OK-01	3	0.460950783
TCGA-V1-A8MG-01	3	0.895935311
TCGA-V1-A8MK-01	3	0.819799268
TCGA-V1-A8ML-01	3	0.91253928
TCGA-V1-A8MM-01	3	0.879434354
TCGA-V1-A8X3-01	3	0.928953735
TCGA-V1-A9OA-01	3	0.873722069
TCGA-V1-A9OF-01	3	0.897262826
TCGA-V1-A9OL-01	3	0.924043165
TCGA-V1-A9OT-01	3	0.919959858
TCGA-V1-A9OY-01	3	0.902353779
TCGA-V1-A9Z8-01	3	0.749003734
TCGA-V1-A9ZR-01	3	0.905764487
TCGA-VN-A88I-01	3	0.833231916
TCGA-VN-A88M-01	3	0.574013333
TCGA-VN-A88O-01	3	0.67740347
TCGA-VN-A88P-01	3	0.924482305
TCGA-VN-A943-01	3	0.892856448
TCGA-VP-A87E-01	3	0.88597015
TCGA-VP-A87J-01	3	0.918746306
TCGA-XA-A8JR-01	3	0.889052912
TCGA-XJ-A9DQ-01	3	0.922534687
TCGA-XK-AAIW-01	3	0.807050866
TCGA-XK-AAJA-01	3	0.684513932
TCGA-XK-AAJT-01	3	0.888643893
TCGA-XK-AAJU-01	3	0.747882879
TCGA-YJ-A8SW-01	3	0.930304101
TCGA-YL-A8SA-01	3	0.886783282
TCGA-YL-A8SI-01	3	0.894266122

TCGA-YL-A8SK-01	3	0.910831826
TCGA-YL-A9WJ-01	3	0.904962311
TCGA-YL-A9WL-01	3	0.917656258
TCGA-ZG-A8QX-01	3	0.913940979
TCGA-ZG-A8QZ-01	3	0.82949899
TCGA-ZG-A9L9-01	3	0.92344047
TCGA-ZG-A9LU-01	3	0.852087617
TCGA-ZG-A9MC-01	3	0.879751221

Table S3 List of 401 core samples with 3 subtypes and silhouette width

Sample name	Subtype	Silhouette width
TCGA-2A-A8VT-01	1	0.526173114
TCGA-2A-A8VV-01	1	0.761408761
TCGA-2A-A8VX-01	1	0.784589591
TCGA-2A-A8W1-01	1	0.814626494
TCGA-CH-5741-01	1	0.833070894
TCGA-CH-5743-01	1	0.630107793
TCGA-CH-5745-01	1	0.779773099
TCGA-CH-5750-01	1	0.465009732
TCGA-CH-5751-01	1	0.787079248
TCGA-CH-5752-01	1	0.803495028
TCGA-CH-5754-01	1	0.789855024
TCGA-CH-5761-01	1	0.735255878
TCGA-CH-5762-01	1	0.580771083
TCGA-CH-5764-01	1	0.757924634
TCGA-CH-5768-01	1	0.852773904
TCGA-CH-5772-01	1	0.815097573
TCGA-CH-5788-01	1	0.764553946
TCGA-CH-5792-01	1	0.760115956
TCGA-EJ-5494-01	1	0.736803098
TCGA-EJ-5498-01	1	0.698749531
TCGA-EJ-5499-01	1	0.722743966
TCGA-EJ-5504-01	1	0.628599417
TCGA-EJ-5507-01	1	0.710269348
TCGA-EJ-5509-01	1	0.495883391
TCGA-EJ-5512-01	1	0.541798607
TCGA-EJ-5514-01	1	0.605008517
TCGA-EJ-5521-01	1	0.720271037
TCGA-EJ-5525-01	1	0.802612271
TCGA-EJ-5526-01	1	0.698735997
TCGA-EJ-5530-01	1	0.756849468
TCGA-EJ-5531-01	1	0.696355424
TCGA-EJ-7115-01	1	0.692849141
TCGA-EJ-7123-01	1	0.598689964
TCGA-EJ-7315-01	1	0.747521436
TCGA-EJ-7318-01	1	0.732466025
TCGA-EJ-7321-01	1	0.577058384
TCGA-EJ-7328-01	1	0.808606669
TCGA-EJ-7331-01	1	0.819773088
TCGA-EJ-7781-01	1	0.828412912

TCGA-EJ-7784-01	1	0.801372621
TCGA-EJ-7785-01	1	0.822014272
TCGA-EJ-7789-01	1	0.828031024
TCGA-EJ-7793-01	1	0.631523625
TCGA-EJ-7797-01	1	0.715373117
TCGA-EJ-8469-01	1	0.785998856
TCGA-EJ-8472-01	1	0.792585273
TCGA-EJ-8474-01	1	0.667994982
TCGA-EJ-A46E-01	1	0.291166713
TCGA-EJ-A46F-01	1	0.477463468
TCGA-EJ-A65D-01	1	0.699949245
TCGA-EJ-A65E-01	1	0.338565301
TCGA-EJ-A7NH-01	1	0.715415914
TCGA-EJ-A7NJ-01	1	0.573419307
TCGA-EJ-A7NN-01	1	0.42641299
TCGA-EJ-AB27-01	1	0.317525273
TCGA-FC-A5OB-01	1	0.81187667
TCGA-FC-A66V-01	1	0.700263715
TCGA-FC-A6HD-01	1	0.847585006
TCGA-FC-A8O0-01	1	0.737018037
TCGA-G9-6329-01	1	0.538908582
TCGA-G9-6332-01	1	0.664704593
TCGA-G9-6333-01	1	0.771550988
TCGA-G9-6336-01	1	0.664234814
TCGA-G9-6361-01	1	0.599792317
TCGA-G9-6365-01	1	0.595757564
TCGA-G9-6367-01	1	0.688830731
TCGA-G9-6369-01	1	0.807992971
TCGA-G9-6370-01	1	0.729555102
TCGA-G9-6377-01	1	0.824164157
TCGA-G9-6385-01	1	0.762483866
TCGA-G9-6498-01	1	0.394514762
TCGA-G9-6499-01	1	0.786812648
TCGA-G9-7509-01	1	0.80170814
TCGA-G9-7510-01	1	0.690922217
TCGA-G9-7521-01	1	0.730222854
TCGA-G9-7525-01	1	0.718176446
TCGA-G9-A9S0-01	1	0.68437256
TCGA-G9-A9S4-01	1	0.602959773
TCGA-HC-7078-01	1	0.750331511
TCGA-HC-7212-01	1	0.629298391

TCGA-HC-7213-01	1	0.787634601
TCGA-HC-7230-01	1	0.709962077
TCGA-HC-7231-01	1	0.794906463
TCGA-HC-7232-01	1	0.700182399
TCGA-HC-7233-01	1	0.760911064
TCGA-HC-7737-01	1	0.718066991
TCGA-HC-7738-01	1	0.756931021
TCGA-HC-7740-01	1	0.795338437
TCGA-HC-7742-01	1	0.731926121
TCGA-HC-7745-01	1	0.741118028
TCGA-HC-8216-01	1	0.62768948
TCGA-HC-8256-01	1	0.812243851
TCGA-HC-8260-01	1	0.590517318
TCGA-HC-8261-01	1	0.749810526
TCGA-HC-8262-01	1	0.708440529
TCGA-HC-8264-01	1	0.675311365
TCGA-HC-8265-01	1	0.754079949
TCGA-HC-A48F-01	1	0.752321183
TCGA-HC-A631-01	1	0.638558665
TCGA-HC-A632-01	1	0.574259587
TCGA-HC-A6AP-01	1	0.670719067
TCGA-HC-A76W-01	1	0.240880827
TCGA-HC-A8D0-01	1	0.468478945
TCGA-HC-A9TE-01	1	0.545882911
TCGA-HC-A9TH-01	1	0.850469597
TCGA-HI-7171-01	1	0.761979597
TCGA-J4-8200-01	1	0.76722962
TCGA-J4-A67L-01	1	0.702594131
TCGA-J4-A67M-01	1	0.746901902
TCGA-J4-A67R-01	1	0.763615873
TCGA-J4-A6G3-01	1	0.856244947
TCGA-J4-A83I-01	1	0.709074545
TCGA-J4-AAU2-01	1	0.450902939
TCGA-J9-A52B-01	1	0.721992125
TCGA-J9-A8CK-01	1	0.668952063
TCGA-J9-A8CL-01	1	0.749398088
TCGA-J9-A8CN-01	1	0.823898724
TCGA-KC-A4BL-01	1	0.741282434
TCGA-KC-A4BR-01	1	0.531156799
TCGA-KC-A7F6-01	1	0.792504118
TCGA-KC-A7FA-01	1	0.594602158

TCGA-KC-A7FD-01	1	0.818851211
TCGA-KK-A59X-01	1	0.651854319
TCGA-KK-A59Z-01	1	0.310680089
TCGA-KK-A5A1-01	1	0.827499968
TCGA-KK-A6DY-01	1	0.810286708
TCGA-KK-A6E2-01	1	0.706391001
TCGA-KK-A6E8-01	1	0.216888442
TCGA-KK-A7AP-01	1	0.837811808
TCGA-KK-A7AU-01	1	0.640015944
TCGA-KK-A7AZ-01	1	0.785258822
TCGA-KK-A7B0-01	1	0.560387484
TCGA-KK-A7B2-01	1	0.788213175
TCGA-KK-A7B3-01	1	0.618514174
TCGA-KK-A7B4-01	1	0.704506374
TCGA-KK-A8IC-01	1	0.436900055
TCGA-KK-A8IG-01	1	0.707502944
TCGA-KK-A8IH-01	1	0.538825358
TCGA-KK-A8II-01	1	0.815179382
TCGA-KK-A8IK-01	1	0.786365349
TCGA-M7-A71Y-01	1	0.699263273
TCGA-M7-A722-01	1	0.765293491
TCGA-M7-A724-01	1	0.621242655
TCGA-QU-A6IM-01	1	0.70269211
TCGA-QU-A6IN-01	1	0.648654978
TCGA-QU-A6IP-01	1	0.649999707
TCGA-TP-A8TT-01	1	0.80184369
TCGA-TP-A8TV-01	1	0.62615959
TCGA-V1-A8MJ-01	1	0.698887439
TCGA-V1-A8MU-01	1	0.718989326
TCGA-V1-A8WN-01	1	0.460685019
TCGA-V1-A8WV-01	1	0.536585723
TCGA-V1-A8WW-01	1	0.788611964
TCGA-V1-A9O5-01	1	0.638394345
TCGA-V1-A9O9-01	1	0.812257577
TCGA-V1-A9OQ-01	1	0.834670384
TCGA-V1-A9OX-01	1	0.843324784
TCGA-V1-A9ZG-01	1	0.777860146
TCGA-V1-A9ZI-01	1	0.707678713
TCGA-VN-A88L-01	1	0.374446876
TCGA-VN-A88Q-01	1	0.718209398
TCGA-VN-A88R-01	1	0.767397755

TCGA-VP-A872-01	1	0.751138683
TCGA-VP-A875-01	1	0.846232802
TCGA-VP-A876-01	1	0.781827199
TCGA-VP-A879-01	1	0.764418738
TCGA-VP-A87B-01	1	0.624261948
TCGA-VP-A87D-01	1	0.509107691
TCGA-VP-A87H-01	1	0.728572103
TCGA-VP-A87K-01	1	0.735809744
TCGA-VP-AA1N-01	1	0.783705576
TCGA-X4-A8KS-01	1	0.565186207
TCGA-XJ-A83H-01	1	0.353677631
TCGA-XJ-A9DI-01	1	0.735143567
TCGA-XJ-A9DX-01	1	0.6334439
TCGA-XK-AAIR-01	1	0.268133507
TCGA-XK-AAIV-01	1	0.629134143
TCGA-XK-AAJ3-01	1	0.636313773
TCGA-XK-AAJR-01	1	0.349206447
TCGA-XK-AAK1-01	1	0.650932808
TCGA-YL-A8HK-01	1	0.739518755
TCGA-YL-A8HL-01	1	0.747302729
TCGA-YL-A8HM-01	1	0.721227452
TCGA-YL-A8HO-01	1	0.821352606
TCGA-YL-A8SB-01	1	0.780528761
TCGA-YL-A8SJ-01	1	0.703500786
TCGA-YL-A8SQ-01	1	0.721105713
TCGA-YL-A8SR-01	1	0.710781051
TCGA-YL-A9WH-01	1	0.703171118
TCGA-YL-A9WK-01	1	0.65814183
TCGA-YL-A9WX-01	1	0.668175457
TCGA-YL-A9WY-01	1	0.735453249
TCGA-ZG-A9KY-01	1	0.792421313
TCGA-ZG-A9L0-01	1	0.555666902
TCGA-ZG-A9L1-01	1	0.614911111
TCGA-ZG-A9L4-01	1	0.827209501
TCGA-ZG-A9LB-01	1	0.723398109
TCGA-ZG-A9LM-01	1	0.687732469
TCGA-ZG-A9LZ-01	1	0.572916018
TCGA-ZG-A9M4-01	1	0.700961106
TCGA-ZG-A9N3-01	1	0.684849974
TCGA-ZG-A9ND-01	1	0.551549402
TCGA-ZG-A9NI-01	1	0.707424198

TCGA-2A-A8VO-01	2	0.205632777
TCGA-CH-5738-01	2	0.492885668
TCGA-CH-5765-01	2	0.387396377
TCGA-CH-5789-01	2	0.421172472
TCGA-CH-5794-01	2	0.492400301
TCGA-EJ-5508-01	2	0.297054453
TCGA-EJ-5519-01	2	0.529477836
TCGA-EJ-A46G-01	2	0.233101435
TCGA-EJ-A6RC-01	2	0.525022666
TCGA-EJ-A8FN-01	2	0.310283708
TCGA-G9-6354-01	2	0.25310173
TCGA-G9-6364-01	2	0.271388877
TCGA-G9-7522-01	2	0.310017741
TCGA-G9-A9S7-01	2	0.261422937
TCGA-HC-7080-01	2	0.374888132
TCGA-HC-7744-01	2	0.280160044
TCGA-HC-8257-01	2	0.294104991
TCGA-HC-A6AL-01	2	0.247795138
TCGA-HI-7169-01	2	0.236229556
TCGA-HI-7170-01	2	0.204768027
TCGA-J4-A67Q-01	2	0.458232626
TCGA-J4-A6M7-01	2	0.337281328
TCGA-J9-A8CM-01	2	0.433528791
TCGA-KK-A6E1-01	2	0.318284243
TCGA-KK-A6E6-01	2	0.363082927
TCGA-KK-A6E7-01	2	0.246018041
TCGA-KK-A7AY-01	2	0.361987014
TCGA-KK-A8I7-01	2	0.255780699
TCGA-KK-A8IA-01	2	0.328193744
TCGA-KK-A8ID-01	2	0.205103272
TCGA-KK-A8IF-01	2	0.313025546
TCGA-KK-A8IJ-01	2	0.246077419
TCGA-M7-A720-01	2	0.215083955
TCGA-QU-A6IL-01	2	0.514483407
TCGA-QU-A6IO-01	2	0.382480979
TCGA-V1-A8WL-01	2	0.26505927
TCGA-V1-A9Z9-01	2	0.266341351
TCGA-V1-A9ZK-01	2	0.394795451
TCGA-VN-A88K-01	2	0.240604497
TCGA-VP-A878-01	2	0.209600338
TCGA-X4-A8KQ-01	2	0.224824156

TCGA-XJ-A83G-01	2	0.306871103
TCGA-XJ-A9DK-01	2	0.323535969
TCGA-XQ-A8TB-01	2	0.269311276
TCGA-Y6-A9XI-01	2	0.406143405
TCGA-YL-A8S9-01	2	0.380399146
TCGA-YL-A8SC-01	2	0.505404491
TCGA-YL-A8SF-01	2	0.242686406
TCGA-YL-A8SL-01	2	0.335283244
TCGA-ZG-A8QW-01	2	0.241042013
TCGA-ZG-A9L5-01	2	0.272043782
TCGA-ZG-A9LN-01	2	0.318596801
TCGA-ZG-A9LS-01	2	0.261309236
TCGA-ZG-A9LY-01	2	0.291922187
TCGA-2A-A8W3-01	3	0.920406902
TCGA-2A-AAYO-01	3	0.852860937
TCGA-2A-AAYU-01	3	0.742661387
TCGA-CH-5739-01	3	0.455497123
TCGA-CH-5744-01	3	0.927623982
TCGA-CH-5746-01	3	0.829964069
TCGA-CH-5753-01	3	0.785950532
TCGA-CH-5763-01	3	0.686118414
TCGA-CH-5766-01	3	0.320111528
TCGA-CH-5767-01	3	0.766558095
TCGA-CH-5790-01	3	0.891161949
TCGA-EJ-5497-01	3	0.346541848
TCGA-EJ-5502-01	3	0.858401177
TCGA-EJ-5510-01	3	0.737867857
TCGA-EJ-5515-01	3	0.908419048
TCGA-EJ-5516-01	3	0.851466887
TCGA-EJ-5517-01	3	0.921928367
TCGA-EJ-5524-01	3	0.926032544
TCGA-EJ-5532-01	3	0.921556464
TCGA-EJ-5542-01	3	0.886561639
TCGA-EJ-7125-01	3	0.929055085
TCGA-EJ-7312-01	3	0.909800917
TCGA-EJ-7314-01	3	0.930090786
TCGA-EJ-7317-01	3	0.871498547
TCGA-EJ-7327-01	3	0.917883472
TCGA-EJ-7330-01	3	0.745237344
TCGA-EJ-7782-01	3	0.905359991
TCGA-EJ-7788-01	3	0.928949084

TCGA-EJ-7792-01	3	0.82404122
TCGA-EJ-7794-01	3	0.900372364
TCGA-EJ-8468-01	3	0.387787637
TCGA-EJ-8470-01	3	0.914813049
TCGA-EJ-A46D-01	3	0.918852127
TCGA-EJ-A46I-01	3	0.92986405
TCGA-EJ-A65G-01	3	0.821185212
TCGA-EJ-A65J-01	3	0.897598163
TCGA-EJ-A65M-01	3	0.906049366
TCGA-EJ-A6RA-01	3	0.931054079
TCGA-EJ-A7NG-01	3	0.906371549
TCGA-EJ-A7NK-01	3	0.853837167
TCGA-EJ-A8FO-01	3	0.899829863
TCGA-EJ-A8FP-01	3	0.913138575
TCGA-EJ-AB20-01	3	0.929203316
TCGA-FC-7708-01	3	0.35584207
TCGA-FC-A4JI-01	3	0.843087347
TCGA-G9-6338-01	3	0.88444932
TCGA-G9-6339-01	3	0.930022648
TCGA-G9-6342-01	3	0.756261185
TCGA-G9-6351-01	3	0.293877709
TCGA-G9-6353-01	3	0.904623648
TCGA-G9-6356-01	3	0.884596146
TCGA-G9-6363-01	3	0.242553103
TCGA-G9-6371-01	3	0.927291529
TCGA-G9-6378-01	3	0.636551595
TCGA-G9-6384-01	3	0.92937518
TCGA-G9-6494-01	3	0.91345904
TCGA-G9-6496-01	3	0.927533746
TCGA-G9-7523-01	3	0.922676376
TCGA-H9-7775-01	3	0.928233548
TCGA-H9-A6BX-01	3	0.893207272
TCGA-HC-7079-01	3	0.912615197
TCGA-HC-7209-01	3	0.893429329
TCGA-HC-7210-01	3	0.929752112
TCGA-HC-7211-01	3	0.882805619
TCGA-HC-7747-01	3	0.914718964
TCGA-HC-7748-01	3	0.908042303
TCGA-HC-7752-01	3	0.928204456
TCGA-HC-7818-01	3	0.918465328
TCGA-HC-8213-01	3	0.912684408

TCGA-HC-8259-01	3	0.677942371
TCGA-HC-A6AQ-01	3	0.895976722
TCGA-HC-A6AS-01	3	0.805419347
TCGA-HC-A6HX-01	3	0.461394649
TCGA-HC-A8D1-01	3	0.930957927
TCGA-J4-8198-01	3	0.405720131
TCGA-J4-A67K-01	3	0.917741736
TCGA-J4-A67N-01	3	0.897391236
TCGA-J4-A67O-01	3	0.441635067
TCGA-J4-A67S-01	3	0.749530402
TCGA-J4-A67T-01	3	0.928097775
TCGA-J4-A6G1-01	3	0.885692737
TCGA-J4-A83M-01	3	0.587151061
TCGA-J4-A83N-01	3	0.900990327
TCGA-J4-AATV-01	3	0.877420487
TCGA-J9-A52C-01	3	0.886974562
TCGA-J9-A52D-01	3	0.8996399
TCGA-J9-A52E-01	3	0.923522643
TCGA-J9-A8CP-01	3	0.932045065
TCGA-KC-A4BN-01	3	0.837235244
TCGA-KC-A7F3-01	3	0.677066672
TCGA-KC-A7FE-01	3	0.880948526
TCGA-KK-A59V-01	3	0.918377002
TCGA-KK-A6E0-01	3	0.892603139
TCGA-KK-A6E5-01	3	0.927849429
TCGA-KK-A7AQ-01	3	0.80475159
TCGA-KK-A7AV-01	3	0.85121445
TCGA-KK-A7AW-01	3	0.925093537
TCGA-KK-A7B1-01	3	0.376742711
TCGA-KK-A8I5-01	3	0.886792777
TCGA-KK-A8I8-01	3	0.829178074
TCGA-KK-A8IB-01	3	0.74026911
TCGA-KK-A8IL-01	3	0.918655494
TCGA-KK-A8IM-01	3	0.931393051
TCGA-M7-A71Z-01	3	0.90194116
TCGA-M7-A721-01	3	0.86231902
TCGA-MG-AAMC-01	3	0.798667247
TCGA-SU-A7E7-01	3	0.58096008
TCGA-TK-A8OK-01	3	0.460950783
TCGA-V1-A8MG-01	3	0.895935311
TCGA-V1-A8MK-01	3	0.819799268

TCGA-V1-A8ML-01	3	0.91253928
TCGA-V1-A8MM-01	3	0.879434354
TCGA-V1-A8X3-01	3	0.928953735
TCGA-V1-A9OA-01	3	0.873722069
TCGA-V1-A9OF-01	3	0.897262826
TCGA-V1-A9OL-01	3	0.924043165
TCGA-V1-A9OT-01	3	0.919959858
TCGA-V1-A9OY-01	3	0.902353779
TCGA-V1-A9Z8-01	3	0.749003734
TCGA-V1-A9ZR-01	3	0.905764487
TCGA-VN-A88I-01	3	0.833231916
TCGA-VN-A88M-01	3	0.574013333
TCGA-VN-A88O-01	3	0.67740347
TCGA-VN-A88P-01	3	0.924482305
TCGA-VN-A943-01	3	0.892856448
TCGA-VP-A87E-01	3	0.88597015
TCGA-VP-A87J-01	3	0.918746306
TCGA-XA-A8JR-01	3	0.889052912
TCGA-XJ-A9DQ-01	3	0.922534687
TCGA-XK-AAIW-01	3	0.807050866
TCGA-XK-AAJA-01	3	0.684513932
TCGA-XK-AAJT-01	3	0.888643893
TCGA-XK-AAJU-01	3	0.747882879
TCGA-YJ-A8SW-01	3	0.930304101
TCGA-YL-A8SA-01	3	0.886783282
TCGA-YL-A8SI-01	3	0.894266122
TCGA-YL-A8SK-01	3	0.910831826
TCGA-YL-A9WJ-01	3	0.904962311
TCGA-YL-A9WL-01	3	0.917656258
TCGA-ZG-A8QX-01	3	0.913940979
TCGA-ZG-A8QZ-01	3	0.82949899
TCGA-ZG-A9L9-01	3	0.92344047
TCGA-ZG-A9LU-01	3	0.852087617
TCGA-ZG-A9MC-01	3	0.879751221

Table S4 The biomarker genes for three different molecular subtypes

Gene symbol	Coefficients	Subtype
NFKB1	-12.67722812	1
OR2T10	2.451072377	1
BRCA2	4.395135437	1
OR2A5	7.030826614	1
OR10K2	9.891728763	1
OR4N2	16.07500854	1
HDAC2	16.55091715	1
CREBBP	19.32242332	1
KRIT1	23.42609735	1
OR2G6	26.63854641	1
ADCY2	29.20352508	1
OR10J5	33.47009126	1
OR4X1	35.87233392	1
OR4P4	37.39009525	1
OR5T2	38.64296709	1
MYH14	38.85627107	1
OR10J3	43.00453214	1
ACTG1	43.55185133	1
OR6C76	48.33336361	1
OR2D2	51.61813906	1
KDR	55.24021576	1
NTRK1	55.87428565	1
HRAS	63.94823609	1
OR56A1	65.86566315	1
OR51E1	70.94500095	1
COL1A1	72.64071948	1
ITGA6	76.44198013	1
OR5F1	79.41648207	1
OR5AS1	82.73351343	1
UBC	83.16021967	1
MTOR	83.54940385	1
OR2A2	85.30370239	1
OR5R1	85.7918911	1
SHC1	85.94381352	1
OR1G1	89.38585553	1
OR10J1	92.00088999	1
PIK3CB	93.00868403	1
OR5A1	94.6045341	1

STAT3	96.54136798	1
ANK1	98.66709053	1
IL6	100.4233431	1
IL4	101.1005351	1
AR	101.2147723	1
SP1	101.7178553	1
KAT5	102.7919166	1
BMP4	112.4113919	1
OR7G1	115.7024739	1
OR2AG2	116.4160984	1
OR5D16	120.6899122	1
PIK3CD	122.8536783	1
PXN	123.4788235	1
ITGA2	123.6515299	1
GNAL	125.6701219	1
OR52E4	128.1774253	1
CBL	131.2143373	1
ERBB2	131.6632302	1
OR4S1	134.9297656	1
OR6P1	137.3384069	1
MMP9	144.3281386	1
DNAH8	146.720443	1
OR5B3	146.8853633	1
IGF1R	153.4344537	1
MYB	156.1278189	1
OR5T1	156.8411101	1
PIK3CA	161.8610423	1
CTNNB1	177.7493137	1
VEGFA	181.1600111	1
IL8	185.964571	1
MUC2	192.0040057	1
OR4S2	193.2614992	1
MUC5B	194.4222356	1
SERPINE1	198.3764534	1
TP53	277.3215029	1
UBA52	332.5565838	1
EGF	362.1371866	1
PSEN1	476.549848	1
BCL2	691.1048815	1
YWHAG	1391.703903	1
ALB	1519.02351	1

OR1K1	4.05E-12	2
OR52R1	5.96479188	2
OR5P3	5.995506949	2
OR8D1	8.046868043	2
CDC20	19.11713971	2
OR52M1	20.13691187	2
OR8D2	23.97559808	2
ITGB6	32.33016093	2
PAX5	32.51149754	2
OR2C3	46.1806327	2
OR4A16	53.87388041	2
OR52A5	59.79756917	2
OR10Q1	69.89109026	2
ATM	138.6093227	2
OR9I1	172.0394878	2
OR4C6	174.7920316	2
COL1A2	178.5280655	2
FN1	180.5678302	2
PTEN	194.6960109	2
PRKDC	200.8801849	2
EGFR	212.0005448	2
CHEK2	223.9639634	2
OR1J1	233.9500837	2
YY1	241.8283373	2
OR10R2	242.7171334	2
DNM2	284.1741623	2
XRCC6	290.6482685	2
PTPRC	345.8073367	2
OR5K4	-20782.55662	3
KITLG	-2073.641317	3
UBA52	-908.5334135	3
EGF	-866.219996	3
JAK2	-699.1263273	3
CDC42	-657.5856107	3
RAD51	-539.8055344	3
MUC1	-512.5302135	3
ELANE	-463.0265909	3
COL2A1	-462.5827318	3
OR5B2	-152.5139016	3
BCL2	-106.7232392	3
BDNF	-67.68322231	3

PIK3CG	-54.93572614	3
OR5D13	-35.92206537	3
CHEK2	-35.39741344	3
OR6K2	-23.78702455	3
OR10Q1	-23.40208097	3
BRCA2	-10.85917416	3
PIK3CB	-8.814043274	3
OR4P4	-8.519202764	3
ATM	-3.742817762	3
OR2AP1	-0.179173523	3
OR51J1	-4.03E-11	3
OR51H1P	-2.33E-11	3
OR4C5	-2.06E-11	3
OR2V1	-1.39E-11	3
OR2AJ1	-8.95E-13	3
GSK3A	0.123089489	3
FYN	3.759315932	3
OR10A4	10.56407414	3
OR8K3	12.20220639	3
HDAC3	15.10753992	3
OR4X2	18.08742113	3
BCR	19.38124496	3
OR10H4	21.12963674	3
OR5AR1	21.99814848	3
OR9G4	22.22377333	3
OR6Y1	23.9958307	3
OR6T1	25.18898324	3
OR7A17	29.03911674	3
OR8S1	30.20799378	3
OR7D4	30.29041939	3
OR5K3	32.24347247	3
OR4Q3	38.55608673	3
OR9A2	38.96982887	3
OR6C75	47.94504324	3
OR5P2	51.46932037	3
OR8B8	53.04669656	3
POLR2A	57.96952346	3
OR51D1	58.35215645	3
OR2S2	61.782717	3
ITGAV	61.96826528	3
OR2AK2	66.22407508	3

OR5B17	66.86638869	3
OR6X1	74.95601569	3
HIF1A	75.39173054	3
OR5AK2	135.112495	3
MAX	164.3129761	3
NFKB1	185.3107737	3
PRKCA	255.9489789	3
POU5F1	527.5447042	3
FAM48A	552.2737588	3

Table S5 The KEGG enrichment results for three different molecular subtypes (continue)

Subtype	KEGG ID	Term	Count	Percentage (%)
1	hsa04740	Olfactory transduction	30	37.97
1	hsa05200	Pathways in cancer	26	32.91
1	hsa05215	Prostate cancer	14	17.72
1	hsa04510	Focal adhesion	17	21.52
1	hsa05212	Pancreatic cancer	10	12.66
1	hsa05214	Glioma	9	11.39
1	hsa04722	Neurotrophin signaling pathway	11	13.92
1	hsa05220	Chronic myeloid leukemia	9	11.39
1	hsa05213	Endometrial cancer	8	10.13
1	hsa04012	ErbB signaling pathway	9	11.39
1	hsa05219	Bladder cancer	7	8.86
1	hsa05223	Non-small cell lung cancer	7	8.86
1	hsa05222	Small cell lung cancer	8	10.13
1	hsa05221	Acute myeloid leukemia	7	8.86
1	hsa05218	Melanoma	7	8.86
1	hsa04370	VEGF signaling pathway	7	8.86
1	hsa05210	Colorectal cancer	7	8.86
1	hsa04062	Chemokine signaling pathway	10	12.66
1	hsa04210	Apoptosis	7	8.86
1	hsa05211	Renal cell carcinoma	6	7.59
1	hsa04810	Regulation of actin cytoskeleton	10	12.66
1	hsa04660	T cell receptor signaling pathway	7	8.86
1	hsa04150	mTOR signaling pathway	5	6.33
1	hsa04630	Jak-STAT signaling pathway	8	10.13
1	hsa04670	Leukocyte transendothelial migration	7	8.86
1	hsa05216	Thyroid cancer	4	5.06
1	hsa04910	Insulin signaling pathway	7	8.86
1	hsa04620	Toll-like receptor signaling pathway	6	7.59
2	hsa04740	Olfactory transduction	14	50
3	hsa04740	Olfactory transduction	26	41.27
3	hsa05200	Pathways in cancer	14	22.22
3	hsa05212	Pancreatic cancer	7	11.11
3	hsa04510	Focal adhesion	9	14.29
3	hsa05222	Small cell lung cancer	6	9.52

Table S5 The KEGG enrichment results for three different molecular subtypes (continue)

P-value	Benjamini corrected P-value
1.74E-15	1.55E-13
3.21E-13	1.40E-11
1.25E-10	3.61E-09
7.38E-09	1.61E-07
4.48E-07	7.79E-06
1.82E-06	2.64E-05
5.95E-06	6.47E-05
6.95E-06	6.72E-05
5.65E-06	7.02E-05
2.11E-05	1.67E-04
1.94E-05	1.69E-04
8.41E-05	6.09E-04
1.35E-04	8.38E-04
1.26E-04	8.44E-04
3.88E-04	2.25E-03
5.22E-04	2.84E-03
9.57E-04	4.61E-03
9.48E-04	4.84E-03
1.15E-03	5.26E-03
2.59E-03	1.07E-02
2.51E-03	1.09E-02
3.48E-03	1.37E-02
5.42E-03	1.87E-02
5.11E-03	1.92E-02
5.38E-03	1.94E-02
7.07E-03	2.34E-02
1.02E-02	3.25E-02
1.22E-02	3.75E-02
4.09E-09	1.64E-07
3.36E-16	2.50E-14
8.32E-06	3.12E-04
5.64E-05	1.41E-03
5.78E-04	1.08E-02
1.18E-03	1.75E-02

Table S6 The GO enrichment results for three different molecular subtypes (continue)

Subtype	ID	Term
1	GO:0007608	Sensory Perception Of Smell
1	GO:0004984	Olfactory Receptor Activity
1	GO:0007166	Cell Surface Receptor Linked Signal Transduction
1	GO:0007606	Sensory Perception Of Chemical Stimulus
1	GO:0050877	Neurological System Process
1	GO:0050890	Cognition
1	GO:0007600	Sensory Perception
1	GO:0007186	G-Protein Coupled Receptor Protein Signaling Pathway
1	GO:0005886	Plasma Membrane
1	GO:0010557	Positive Regulation Of Macromolecule Biosynthetic Process
1	GO:0031328	Positive Regulation Of Cellular Biosynthetic Process
1	GO:0009891	Positive Regulation Of Biosynthetic Process
1	GO:0010604	Positive Regulation Of Macromolecule Metabolic Process
1	GO:0043066	Negative Regulation Of Apoptosis
1	GO:0060548	Negative Regulation Of Cell Death
1	GO:0043069	Negative Regulation Of Programmed Cell Death
1	GO:0042127	Regulation Of Cell Proliferation
1	GO:0045935	Positive Regulation Of Nucleobase-Nucleoside-Nucleotide And Nucleic Acid Metabolic Process
1	GO:0009719	Response To Endogenous Stimulus
1	GO:0030097	Hemopoiesis
1	GO:0051173	Positive Regulation Of Nitrogen Compound Metabolic Process
1	GO:0009725	Response To Hormone Stimulus
1	GO:0048534	Hemopoietic Or Lymphoid Organ Development
1	GO:0002520	Immune System Development
1	GO:0010941	Regulation Of Cell Death
1	GO:0008284	Positive Regulation Of Cell Proliferation
1	GO:0045944	Positive Regulation Of Transcription From Rna Polymerase Ii Promoter
1	GO:0040017	Positive Regulation Of Locomotion
1	GO:0042981	Regulation Of Apoptosis
1	GO:0010647	Positive Regulation Of Cell Communication
1	GO:0043067	Regulation Of Programmed Cell Death
1	GO:0045941	Positive Regulation Of Transcription
1	GO:0009991	Response To Extracellular Stimulus
1	GO:0010628	Positive Regulation Of Gene Expression
1	GO:0044093	Positive Regulation Of Molecular Function
1	GO:0007169	Transmembrane Receptor Protein Tyrosine Kinase Signaling Pathway
1	GO:0014070	Response To Organic Cyclic Substance

1	GO:0032270	Positive Regulation Of Cellular Protein Metabolic Process
1	GO:0048871	Multicellular Organismal Homeostasis
1	GO:0051247	Positive Regulation Of Protein Metabolic Process
1	GO:0030335	Positive Regulation Of Cell Migration
1	GO:0010033	Response To Organic Substance
1	GO:0040012	Regulation Of Locomotion
1	GO:0031667	Response To Nutrient Levels
1	GO:0045893	Positive Regulation Of Transcription-Dna-Dependent
1	GO:0051254	Positive Regulation Of Rna Metabolic Process
1	GO:0042592	Homeostatic Process
1	GO:0019220	Regulation Of Phosphate Metabolic Process
1	GO:0051174	Regulation Of Phosphorus Metabolic Process
1	GO:0007242	Intracellular Signaling Cascade
1	GO:0051272	Positive Regulation Of Cell Motion
1	GO:0050921	Positive Regulation Of Chemotaxis
1	GO:0042327	Positive Regulation Of Phosphorylation
1	GO:0010562	Positive Regulation Of Phosphorus Metabolic Process
1	GO:0045937	Positive Regulation Of Phosphate Metabolic Process
1	GO:0007167	Enzyme Linked Receptor Protein Signaling Pathway
1	GO:0050920	Regulation Of Chemotaxis
1	GO:0001894	Tissue Homeostasis
1	GO:0048754	Branching Morphogenesis Of A Tube
1	GO:0007569	Cell Aging
1	GO:0043085	Positive Regulation Of Catalytic Activity
1	GO:0048520	Positive Regulation Of Behavior
1	GO:0030334	Regulation Of Cell Migration
1	GO:0001763	Morphogenesis Of A Branching Structure
1	GO:0042325	Regulation Of Phosphorylation
1	GO:0032268	Regulation Of Cellular Protein Metabolic Process
1	GO:0006928	Cell Motion
1	GO:0035239	Tube Morphogenesis
1	GO:0031401	Positive Regulation Of Protein Modification Process
1	GO:0002521	Leukocyte Differentiation
1	GO:0048545	Response To Steroid Hormone Stimulus
1	GO:0051960	Regulation Of Nervous System Development
1	GO:0051270	Regulation Of Cell Motion
1	GO:0032583	Regulation Of Gene-Specific Transcription
1	GO:0048732	Gland Development
1	GO:0030155	Regulation Of Cell Adhesion
1	GO:0050795	Regulation Of Behavior
1	GO:0001934	Positive Regulation Of Protein Amino Acid Phosphorylation

1	GO:0050927	Positive Regulation Of Positive Chemotaxis
1	GO:0050926	Regulation Of Positive Chemotaxis
1	GO:0006916	Anti-Apoptosis
1	GO:0008283	Cell Proliferation
1	GO:0030182	Neuron Differentiation
1	GO:0001775	Cell Activation
1	GO:0005942	Phosphoinositide 3-Kinase Complex
1	GO:0043434	Response To Peptide Hormone Stimulus
1	GO:0035295	Tube Development
1	GO:0009967	Positive Regulation Of Signal Transduction
1	GO:0031399	Regulation Of Protein Modification Process
1	GO:0032101	Regulation Of Response To External Stimulus
1	GO:0060249	Anatomical Structure Homeostasis
1	GO:0048742	Regulation Of Skeletal Muscle Fiber Development
1	GO:0019932	Second-Messenger-Mediated Signaling
1	GO:0006275	Regulation Of Dna Replication
1	GO:0022602	Ovulation Cycle Process
1	GO:0008406	Gonad Development
1	GO:0001932	Regulation Of Protein Amino Acid Phosphorylation
1	GO:0051240	Positive Regulation Of Multicellular Organismal Process
1	GO:0051052	Regulation Of Dna Metabolic Process
1	GO:0008585	Female Gonad Development
1	GO:0048167	Regulation Of Synaptic Plasticity
1	GO:0032103	Positive Regulation Of Response To External Stimulus
1	GO:0001568	Blood Vessel Development
1	GO:0048641	Regulation Of Skeletal Muscle Tissue Development
1	GO:0001944	Vasculature Development
1	GO:0042698	Ovulation Cycle
1	GO:0040007	Growth
1	GO:0046660	Female Sex Differentiation
1	GO:0048592	Eye Morphogenesis
1	GO:0046545	Development Of Primary Female Sexual Characteristics
1	GO:0044092	Negative Regulation Of Molecular Function
1	GO:0003006	Reproductive Developmental Process
1	GO:0048608	Reproductive Structure Development
1	GO:0051153	Regulation Of Striated Muscle Cell Differentiation
1	GO:0045137	Development Of Primary Sexual Characteristics
1	GO:0006357	Regulation Of Transcription From Rna Polymerase Ii Promoter
1	GO:0043086	Negative Regulation Of Catalytic Activity
1	GO:0060284	Regulation Of Cell Development
1	GO:0045165	Cell Fate Commitment

1	GO:0007584	Response To Nutrient
1	GO:0051147	Regulation Of Muscle Cell Differentiation
1	GO:0006468	Protein Amino Acid Phosphorylation
1	GO:0048589	Developmental Growth
1	GO:0043193	Positive Regulation Of Gene-Specific Transcription
1	GO:0051969	Regulation Of Transmission Of Nerve Impulse
1	GO:0001525	Angiogenesis
1	GO:0048015	Phosphoinositide-Mediated Signaling
1	GO:0001541	Ovarian Follicle Development
1	GO:0007160	Cell-Matrix Adhesion
1	GO:0032965	Regulation Of Collagen Biosynthetic Process
1	GO:0007548	Sex Differentiation
1	GO:0043523	Regulation Of Neuron Apoptosis
1	GO:0044445	Cytosolic Part
1	GO:0031644	Regulation Of Neurological System Process
1	GO:0051098	Regulation Of Binding
1	GO:0030099	Myeloid Cell Differentiation
1	GO:0048593	Camera-Type Eye Morphogenesis
1	GO:0007423	Sensory Organ Development
1	GO:0010712	Regulation Of Collagen Metabolic Process
1	GO:0010551	Regulation Of Specific Transcription From Rna Polymerase Ii Promoter
1	GO:0031589	Cell-Substrate Adhesion
1	GO:0030324	Lung Development
1	GO:0030183	B Cell Differentiation
1	GO:0048872	Homeostasis Of Number Of Cells
1	GO:0045321	Leukocyte Activation
1	GO:0021700	Developmental Maturation
1	GO:0043406	Positive Regulation Of Map Kinase Activity
1	GO:0030323	Respiratory Tube Development
1	GO:0030098	Lymphocyte Differentiation
1	GO:0016202	Regulation Of Striated Muscle Tissue Development
1	GO:0043627	Response To Estrogen Stimulus
1	GO:0010605	Negative Regulation Of Macromolecule Metabolic Process
1	GO:0048634	Regulation Of Muscle Development
1	GO:0043524	Negative Regulation Of Neuron Apoptosis
1	GO:0007176	Regulation Of Epidermal Growth Factor Receptor Activity
1	GO:0002687	Positive Regulation Of Leukocyte Migration
1	GO:0046854	Phosphoinositide Phosphorylation
1	GO:0046934	Phosphatidylinositol-4-5-Bisphosphate 3-Kinase Activity
1	GO:0048666	Neuron Development
1	GO:0010942	Positive Regulation Of Cell Death

1	GO:0060541	Respiratory System Development
1	GO:0001889	Liver Development
1	GO:0040008	Regulation Of Growth
1	GO:0046834	Lipid Phosphorylation
1	GO:0007568	Aging
1	GO:0042476	Odontogenesis
1	GO:0045859	Regulation Of Protein Kinase Activity
1	GO:0051054	Positive Regulation Of Dna Metabolic Process
1	GO:0044246	Regulation Of Multicellular Organismal Metabolic Process
1	GO:0043549	Regulation Of Kinase Activity
1	GO:0008285	Negative Regulation Of Cell Proliferation
1	GO:0010469	Regulation Of Receptor Activity
2	GO:0004984	Olfactory Receptor Activity
2	GO:0007608	Sensory Perception Of Smell
2	GO:0007606	Sensory Perception Of Chemical Stimulus
2	GO:0007166	Cell Surface Receptor Linked Signal Transduction
2	GO:0050890	Cognition
2	GO:0007600	Sensory Perception
2	GO:0050877	Neurological System Process
2	GO:0007186	G-Protein Coupled Receptor Protein Signaling Pathway
2	GO:0005886	Plasma Membrane
2	GO:0010212	Response To Ionizing Radiation
2	GO:0009314	Response To Radiation
3	GO:0004984	Olfactory Receptor Activity
3	GO:0007608	Sensory Perception Of Smell
3	GO:0007606	Sensory Perception Of Chemical Stimulus
3	GO:0050890	Cognition
3	GO:0007600	Sensory Perception
3	GO:0050877	Neurological System Process
3	GO:0007186	G-Protein Coupled Receptor Protein Signaling Pathway
3	GO:0007166	Cell Surface Receptor Linked Signal Transduction
3	GO:0005886	Plasma Membrane
3	GO:0016021	Integral To Membrane
3	GO:0031224	Intrinsic To Membrane
3	GO:0008629	Induction Of Apoptosis By Intracellular Signals
3	GO:0051051	Negative Regulation Of Transport

Table S6 The GO enrichment results for three different molecular subtypes (continue)

Count	Percentage (%)	P-value	Benjamini corrected P-value
31	39.24	3.71E-25	5.86E-22
30	37.97	2.28E-23	4.93E-21
50	63.29	9.97E-24	5.25E-21
31	39.24	7.72E-24	6.10E-21
39	49.37	5.88E-20	1.86E-17
35	44.3	5.65E-20	2.23E-17
33	41.77	2.11E-19	5.55E-17
34	43.04	3.40E-16	7.53E-14
51	64.56	2.56E-10	5.70E-08
20	25.32	3.56E-09	7.04E-07
20	25.32	7.60E-09	1.34E-06
20	25.32	9.62E-09	1.38E-06
22	27.85	9.33E-09	1.47E-06
15	18.99	1.23E-08	1.62E-06
15	18.99	1.52E-08	1.72E-06
15	18.99	1.47E-08	1.78E-06
20	25.32	7.05E-08	6.56E-06
18	22.78	7.04E-08	6.96E-06
15	18.99	6.64E-08	7.00E-06
12	15.19	1.00E-07	8.82E-06
18	22.78	1.11E-07	9.23E-06
14	17.72	1.56E-07	1.23E-05
12	15.19	2.67E-07	2.01E-05
12	15.19	4.84E-07	3.48E-05
19	24.05	6.24E-07	4.11E-05
14	17.72	6.19E-07	4.26E-05
13	16.46	1.29E-06	8.15E-05
8	10.13	1.46E-06	8.90E-05
18	22.78	2.47E-06	1.45E-04
12	15.19	2.71E-06	1.53E-04
18	22.78	2.83E-06	1.54E-04
15	18.99	3.56E-06	1.87E-04
10	12.66	4.66E-06	2.37E-04
15	18.99	5.01E-06	2.48E-04
15	18.99	5.53E-06	2.57E-04
10	12.66	5.39E-06	2.58E-04
8	10.13	6.01E-06	2.71E-04

10	12.66	7.42E-06	3.26E-04
7	8.86	9.21E-06	3.93E-04
10	12.66	1.04E-05	4.33E-04
7	8.86	1.20E-05	4.87E-04
16	20.25	1.30E-05	5.13E-04
9	11.39	1.43E-05	5.50E-04
9	11.39	1.72E-05	6.32E-04
13	16.46	1.68E-05	6.33E-04
13	16.46	1.83E-05	6.57E-04
16	20.25	2.10E-05	6.78E-04
13	16.46	1.99E-05	6.82E-04
13	16.46	1.99E-05	6.82E-04
21	26.58	2.03E-05	6.83E-04
7	8.86	2.09E-05	6.88E-04
5	6.33	2.18E-05	6.88E-04
7	8.86	1.97E-05	6.92E-04
7	8.86	2.34E-05	7.26E-04
7	8.86	2.34E-05	7.26E-04
11	13.92	2.56E-05	7.77E-04
5	6.33	2.86E-05	8.52E-04
6	7.59	3.03E-05	8.87E-04
6	7.59	3.53E-05	1.01E-03
5	6.33	3.69E-05	1.04E-03
13	16.46	3.94E-05	1.09E-03
5	6.33	4.16E-05	1.13E-03
8	10.13	5.26E-05	1.41E-03
6	7.59	6.61E-05	1.74E-03
12	15.19	6.97E-05	1.80E-03
12	15.19	8.12E-05	2.07E-03
12	15.19	8.27E-05	2.07E-03
7	8.86	8.99E-05	2.22E-03
8	10.13	9.95E-05	2.42E-03
7	8.86	1.07E-04	2.55E-03
8	10.13	1.17E-04	2.77E-03
8	10.13	1.17E-04	2.77E-03
8	10.13	1.21E-04	2.77E-03
7	8.86	1.21E-04	2.81E-03
7	8.86	1.26E-04	2.84E-03
7	8.86	1.37E-04	3.04E-03
5	6.33	1.39E-04	3.04E-03
6	7.59	1.59E-04	3.44E-03

4	5.06	1.67E-04	3.57E-03
4	5.06	1.67E-04	3.57E-03
8	10.13	1.81E-04	3.82E-03
11	13.92	1.93E-04	4.01E-03
11	13.92	2.01E-04	4.11E-03
9	11.39	2.41E-04	4.88E-03
4	5.06	4.62E-05	5.14E-03
7	8.86	2.58E-04	5.15E-03
8	10.13	2.71E-04	5.35E-03
9	11.39	2.90E-04	5.65E-03
9	11.39	2.90E-04	5.65E-03
7	8.86	3.07E-04	5.89E-03
6	7.59	3.60E-04	6.84E-03
4	5.06	3.87E-04	7.26E-03
8	10.13	4.05E-04	7.50E-03
5	6.33	4.43E-04	8.11E-03
5	6.33	4.43E-04	8.11E-03
6	7.59	4.64E-04	8.40E-03
7	8.86	4.81E-04	8.61E-03
8	10.13	5.07E-04	8.77E-03
6	7.59	5.03E-04	8.81E-03
5	6.33	5.00E-04	8.84E-03
5	6.33	5.00E-04	8.84E-03
5	6.33	5.00E-04	8.84E-03
8	10.13	5.19E-04	8.88E-03
4	5.06	6.05E-04	1.00E-02
8	10.13	6.00E-04	1.00E-02
5	6.33	5.95E-04	1.01E-02
7	8.86	6.47E-04	1.06E-02
5	6.33	6.65E-04	1.07E-02
5	6.33	6.65E-04	1.07E-02
5	6.33	6.65E-04	1.07E-02
9	11.39	6.62E-04	1.07E-02
8	10.13	7.72E-04	1.23E-02
6	7.59	7.94E-04	1.25E-02
4	5.06	8.11E-04	1.26E-02
6	7.59	8.23E-04	1.27E-02
13	16.46	8.76E-04	1.34E-02
8	10.13	1.07E-03	1.61E-02
7	8.86	1.17E-03	1.74E-02
6	7.59	1.23E-03	1.82E-02

6	7.59	1.27E-03	1.86E-02
4	5.06	1.45E-03	2.10E-02
12	15.19	1.47E-03	2.12E-02
5	6.33	1.58E-03	2.23E-02
5	6.33	1.58E-03	2.23E-02
6	7.59	1.58E-03	2.25E-02
6	7.59	1.63E-03	2.27E-02
5	6.33	1.65E-03	2.29E-02
4	5.06	1.68E-03	2.30E-02
5	6.33	1.72E-03	2.34E-02
3	3.8	1.75E-03	2.35E-02
6	7.59	1.78E-03	2.38E-02
5	6.33	1.80E-03	2.38E-02
7	8.86	3.25E-04	2.39E-02
6	7.59	1.88E-03	2.48E-02
6	7.59	1.88E-03	2.48E-02
5	6.33	2.03E-03	2.64E-02
4	5.06	2.06E-03	2.64E-02
7	8.86	2.05E-03	2.65E-02
3	3.8	2.09E-03	2.65E-02
5	6.33	2.11E-03	2.65E-02
5	6.33	2.45E-03	3.06E-02
5	6.33	2.54E-03	3.15E-02
4	5.06	2.65E-03	3.22E-02
5	6.33	2.64E-03	3.24E-02
7	8.86	2.70E-03	3.26E-02
5	6.33	2.74E-03	3.28E-02
5	6.33	2.83E-03	3.37E-02
5	6.33	2.83E-03	3.37E-02
5	6.33	2.94E-03	3.46E-02
4	5.06	2.97E-03	3.48E-02
5	6.33	3.15E-03	3.60E-02
12	15.19	3.12E-03	3.62E-02
4	5.06	3.15E-03	3.62E-02
4	5.06	3.15E-03	3.62E-02
3	3.8	3.28E-03	3.72E-02
3	3.8	3.28E-03	3.72E-02
3	3.8	3.28E-03	3.72E-02
3	3.8	3.52E-04	3.73E-02
8	10.13	3.36E-03	3.78E-02
9	11.39	3.51E-03	3.84E-02

5	6.33	3.48E-03	3.86E-02
4	5.06	3.51E-03	3.87E-02
8	10.13	3.47E-03	3.88E-02
3	3.8	3.74E-03	3.97E-02
5	6.33	3.72E-03	3.98E-02
4	5.06	3.70E-03	3.99E-02
8	10.13	3.70E-03	4.01E-02
4	5.06	4.10E-03	4.33E-02
3	3.8	4.22E-03	4.42E-02
8	10.13	4.46E-03	4.63E-02
8	10.13	4.73E-03	4.85E-02
3	3.8	4.73E-03	4.87E-02
14	50.00	6.53E-13	7.06E-11
14	50.00	3.90E-13	2.51E-10
14	50.00	1.45E-12	4.68E-10
19	67.86	3.75E-10	6.03E-08
15	53.57	3.05E-10	6.55E-08
14	50.00	1.06E-09	1.36E-07
15	53.57	1.28E-08	1.38E-06
14	50.00	5.52E-08	5.08E-06
21	75.00	7.99E-07	8.39E-05
4	14.29	2.25E-04	1.79E-02
5	17.86	6.23E-04	4.36E-02
35	55.56	4.45E-35	6.18E-33
35	55.56	5.34E-35	5.88E-32
35	55.56	1.87E-33	1.03E-30
39	61.90	2.58E-29	9.45E-27
37	58.73	2.05E-28	5.65E-26
40	63.49	5.41E-26	1.19E-23
38	60.32	1.06E-24	1.95E-22
42	66.67	2.57E-21	4.04E-19
43	68.25	1.80E-11	2.09E-09
40	63.49	6.24E-05	3.62E-03
40	63.49	1.55E-04	5.98E-03
5	7.94	1.07E-04	1.46E-02
6	9.52	3.77E-04	4.51E-02
